# Species delimitation, environmental cline and phylogeny for a new Neotropical genus of Cryptinae (Ichneumonidae)

**DOI:** 10.1371/journal.pone.0237233

**Published:** 2020-10-09

**Authors:** Fernanda A. Supeleto, Bernardo F. Santos, Leandro A. Basilio, Alexandre P. Aguiar

**Affiliations:** 1 Depto de Ciências Biológicas, Universidade Federal do Espírito Santo, Vitória, ES, Brazil; 2 Department of Entomology, National Museum of Natural History, Washington, DC, United States of America; Laboratoire de Biologie du Développement de Villefranche-sur-Mer, FRANCE

## Abstract

A morphologically unusual Cryptini, *Cryptoxenodon* **gen**. **nov**. Supeleto, Santos & Aguiar, is described and illustrated, with a single species, *C*. *metamorphus* **sp**. **nov**. Supeleto, Santos & Aguiar, apparently occurring in two disjunct populations in northern and southeastern South America. The highly dimorphic female and male are described and illustrated. The phylogenetic relationships of the new genus are investigated using a matrix with 308 other species of Cryptini in 182 genera, based on 109 morphological characters and molecular data from seven loci. The analyses clearly support *Cryptoxenodon* **gen**. **nov**. as a distinct genus, closest to *Debilos* Townes and *Diapetimorpha* Viereck. Species limits and definition are investigated, but despite much morphological variation the analyses at the specimen level do not warrant the division of the studied populations into separate species. The considerable morphological variation is explored with principal component analyses of mixed features, and a new procedure is proposed for objective analysis of colors. The relationship of color and structural variation with altitude and latitude is demonstrated and discussed, representing an important case study for Ichneumonidae. Externally, *Cryptoxenodon* **gen**. **nov**. can be recognized mainly by its unusually large mandibles, but other diagnostic features include clypeus wide; sternaulus complete, distinct and crenulate throughout; areolet closed, about as long as pterostigma width; petiole anteriorly with distinct triangular projection on each side, spiracle near posterior 0.25; propodeum without posterior transverse carina; and propodeal apophyses conspicuously projected.

## Introduction

**Fig 1:**
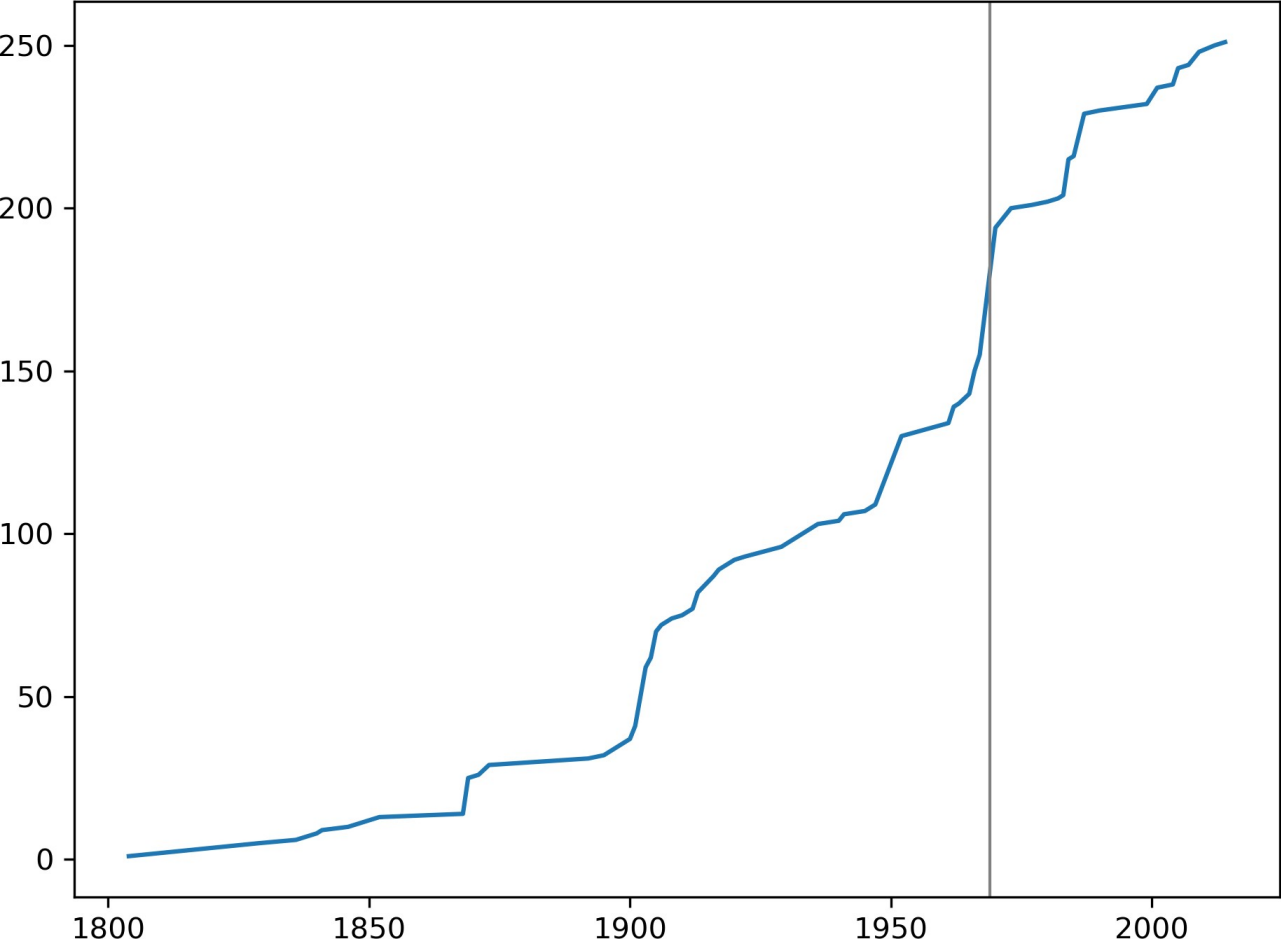
Cumulative number of new genera of Cryptini (Ichneumonidae, Cryptinae) described yearly, up to 2016. Vertical line shows the year before the publication of Townes [7]. Compiled from Yu *et al*. [8].

Ichneumonidae are one of the most diverse and ubiquitous groups of insects, with over 100 thousand estimated species [1]. Contrary to the expectation for most major animal lineages, many groups of ichneumonids seem to be more diverse in temperate regions than in the tropics [2–4]; but see Sääksjärvi [5] and Quicke [6] and references therein. The extremely diverse tribe Cryptini, however, thrives in tropical regions, where they are “the most conspicuous of all ichneumonids” [7]. The group is particularly diverse in the Neotropics, with 1150 species listed in Yu *et al*. [8]. This is about 70% more than in the Oriental (668 spp.) and 80% more than in the Palaearctic (639) regions, and nearly four times more than in the Nearctic region (292). The main framework for the taxonomy of Cryptini, as with most Ichneumonidae, is still the four-volume set of monographs by Townes [9, 7, 10, 11]. In the second and largest volume, Townes describes 40 new genera for Cryptini alone, of which 20 are from the Neotropics. Even after this extensive work, new Cryptini genera are still regularly proposed (Fig 1), having increased from 159 to more than 251 since 1970 (compiled from Yu *et al*. [8]).

Notably, most new genera do not derive from splitting groups of previously known species into separate genera, but from the outright discovery of new species that do not fit into any of the previously known genera. However, these new taxa are often difficult to establish with precision; in part, because most new discoveries fall into large and less characteristic groups of Cryptini, such as Townes’ Goryphina (= *Cryptus* group + *Mesostenus* group, in part, of Santos [12]). Furthermore, the high level of morphological homoplasy observed in Cryptini often obfuscates generic limits. In fact, the most thorough and up-to-date phylogenetic interpretation of Cryptinae [12] has shown that its diversity is as much fascinating as it is morphologically counter-intuitive, leading to a supra-generic classification now based only on informal, albeit monophyletic, genus-groups.

The challenging nature of supra-specific diversity in Cryptini calls for the combination of multiple sources of data and analytical tools to provide a sound assessment of the validity and relationships of proposed new taxa. Accordingly, this work uses an integrative taxonomy approach to propose a new Neotropical genus of Cryptini, to test its placement within the most recent classification for the tribe, and to test and describe its single, but remarkable and broadly occurring species. Our aim is to use this study as a notorious example of the complex external morphological variation in Cryptini, and how it relates to distribution and species delimitation.

## Material and methods

### Repositories

The specimens studied here belong to the following repositories, with the contacted curators between parentheses:

AMNH–American Museum of Natural History, New York (J.M. Carpenter)

CNCI–Canadian National Collection of Insects, Canada (A. Bennett)

INPA–Instituto Nacional de Pesquisas da Amazônia, Manaus, Brazil (M.L. de Oliveira)

MPEG–Museu Paraense Emilio Goeldi, Brazil (O.T. Silveira)

UFES–Universidade Federal do Espírito Santo, Brazil

### Taxonomy and morphology

Morphological terminology and taxonomic conventions follow Santos and Aguiar [13], except that the second trochanter is referred to herein as its more anatomically accurate term, trochantellus; the “posterior transverse carina of mesothoracic venter” is referred to as postpectal carina, for simplicity; and the cell 1+2Rs is called the areolet, as it is standard in ichneumonid systematics literature. The first and subsequent flagellomeres are referred to as f1, f2, f3, etc; the tarsomeres of each leg are referred to, from base to apex, as t1, t2, t3, etc, while first and subsequent metasomal tergites are referred to as T1, T2, T3, etc. When potentially ambiguous, color names are followed by their respective RGB formula, as determined from digital pictures of the studied specimens, according to procedures described by Aguiar [14].

Ratios were rounded to the nearest 0.05, to reflect a realistic degree of precision. Biometric ratios used in descriptions are as follows; the number between brackets corresponds to the index of the respective measurement in the lists provided in the S1 Appendix. **AAW** = areolet minimum width^[1]^ / areolet maximum length (i.e., anterior to posterior)^[2]^; **APH** = areolet maximum length (i.e., anterior to posterior)^[2]^ / pterostigma maximum width^[3]^; **AWH** = areolet maximum width^[4]^ / areolet maximum length (i.e., anterior to posterior)^[2]^; **CHW** = clypeus maximum height^[5]^ / maximum width^[6]^; **FLGM** = number of flagellomeres^[7]^; **FWLG** = fore wing maximum length^[8]^; **HWIA** = hind wing vein 2-1A length^[10]^ / (ibidem + Hind wing vein 2-1A distance from apex to wing margin^[9]^); **HW1C** = hind wing vein Cua length^[11]^ / hind wing crossvein cu-a length^[12]^; **HWHM** = number of hammuli on the hind wing^[13]^; **MELW** = mesoscutum length^[14]^ / width^[15]^; **MLW** = mandible maximum length^[16]^ / maximum width^[17]^; **MWW** = mandible minimum width^[18]^ / maximum width^[17]^; **MSM** = malar space maximum width^[19]^ / mandible maximum width^[17]^; **NLML** = notaulus length^[20]^ / mesoscutum length^[14]^; **OST** = (ovipositor max length^[21]^—pre-sheath ovipositor length^[22]^) / hind tibia max length^[23]^; **OVT** = ovipositor ventral valve, number of teeth^[24]^; **RCUA** = fore wing vein 2Cua, length^[25]^ / fore wing crossvein 2cu-a, length^[26]^; **SPR** = petiole, distance from base to spiracle, in lateral view^[27]^ / (ibidem^[27]^ + petiole, distance from spiracle to apex, in lateral view^[28]^); **SWL** = propodeal spiracle maximum width^[29]^ / maximum length^[30]^; **T1LW** = T1 (petiole) max length^[31]^ / T1 max width, dorsal view^[32]^; **T1WW** = T1 maximum width^[32]^ / T1 min width, dorsal view^[33]^; **T2LW** = T2 max length, dorsal view^[34]^ / T2 max width, dorsal view^[35]^; **T2WW** = T2 max width, dorsal view^[35]^ / T2 min width, dorsal view^[36]^.

### Nomenclatural acts and data availability

**Fig 2:**
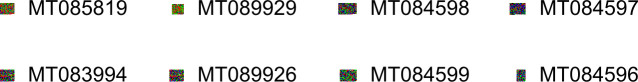
These images encode a total of 5486 base pairs for eight genes (*cytochrome oxidase I*, *16S rRNA*, *18S rRNA*, *28S rRNA*, *arginine kinase exon 1*, *arginine kinase exon 2*, *polymerase II*, and *wingless*), as recovered from *C*. *metamorphus* sp. nov. Images encoded with RGB for ACGT, in groups of 15 bases, as explained in Aguiar [40]. The respective GenBank accession number for each sequence is provided on the right of each image.

The electronic edition of this article conforms to the requirements of the amended International Code of Zoological Nomenclature, and hence the new names contained herein are available under that Code from the electronic edition of this article. This published work and the nomenclatural acts it contains have been registered in ZooBank, the online registration system for the ICZN. The ZooBank LSIDs (Life Science Identifiers) can be resolved and the associated information viewed through any standard web browser by appending the LSID to the prefix “http://zoobank.org/”. The LSID for this publication is: urn:lsid:zoobank.org:pub: B4F31941-37D5-4D48-B007-0A388C08063B. The electronic edition of this work was published in a journal with an ISSN and has been archived and is available from the following digital repository: https://journals.plos.org/plosone/article?id=10.1371/journal.pone.0237233. All original data used to generate this work is provided as (S1–S3 Files) and as molecular data both registered in GenBank (Accession numbers MT083994, MT084596, MT084597, MT084598, MT084599, MT085819, MT089926, MT089929, MT085820, MT085821) and provided directly in the body of the work (Fig 2). All used images are available as a Morphobank project, under the permalink http://morphobank.org/permalink/?P3669.

### Phylogenetic placement

In order to test the validity and phylogenetic placement of the proposed new genus, one specimen (“DNA voucher” in Paratypes list) was included in the phylogenetic matrix of Santos [12], comprising 370 species of Ichneumonidae, including 308 species in 182 genera of Cryptini. Seven molecular loci were sequenced using the exact same primers and protocols as in Santos [12]. All morphological characters used by Santos (op. cit.) were scored for the new taxon, checking all available females.

Regarding the morphological matrix, a warning (item 1 below) and a few corrections (2–5) for the character-set and matrix of Santos [12] must be considered to allow its precise interpretation and usage herein:

1. The scoring in the TNT data matrix provided as supplementary material S4 in Santos [12] presents character-states starting from 1, but in the character-set (Appendix S3 of that work) all character-states start with 0.
2. Character 9 in the data matrix is supernumerary, and therefore *does not* correspond to character 9 in the character-set. It appears in the data matrix scored as [?] (= unknown) for all taxa.
3. In the published data matrix [12], characters 51, 52 and 53 (starting from 1; numbers assuming character 9 above was already removed) must be reordered as 53, 51, 52, respectively, for precise correspondence with characters 51–53 described in the character-set.
4. For character 55, description of state [3] (starting from 0) is missing, and should read “[3] fused with posterior transverse carina”. It was observed only for *Polyaulon* Förster.
5. Comments for character 98 in the character-set mention “State 3” but this is a typo; it should read “State 2”.

Maximum-likelihood analyses were conducted with RAxML v8.2 [15], with the dataset partitioned by locus and using the GTR+ Γ+I model (as indicated by model testing in the original work). Clade support was assessed with 100 rapid bootstrap replicates.

### Species delimitation

To objectively test if the observed intra-specific morphological variation would support more than one species, 24 varying features recognized among examined specimens (Table 1) were coded into a matrix and evaluated with a parsimony analysis using TNT v1.5 [16, 17]. All specimens with some variation for the selected characters were included in the analyses. A species of *Diapetimorpha* Viereck, generally similar and from the same type locality as the new taxon, was used as outgroup. The key objective was to provide a precise and reproducible interpretation of the data, based on the most parsimonious solution for complex, overlapping morphological variations of a considerable number of specimens, which would otherwise be highly subjective to judge. The proposed ordering for character-states was hypothesized from the character analyses [18] of the tree obtained with all states first ran as non-additive. Based on that result, characters 0, 2, 3, 5, 6, 11–15, 17 and 21–23 were run as additive, and all others as non-additive (Table 1). Searches were performed using implied weighting [16]. The constant of concavity (K) was defined by the algorithm setk.run, written by Salvador Arias (Instituto Miguel Lillo, San Miguel de Tucuman, Argentina) according to the rationale of Goloboff et al. [19]. Searches were performed with Ratchet (1000 iterations), sect:slack 40, random seed 11, and hold 20000; all other variables remained set to default values.

**Table 1:** Character coding used in the analyses of morphological variation. Coding reflects all relevant variation observed for females of *Cryptoxenodon metamorphus* sp. nov.

N	Ri	C	Characters and States
0	100	✓	Mandible and clypeus: [0] lighter than head, goldenrod or caramel; [1] mostly dark or mostly darkened, concolorous with head.
1	U	✓	Mandible dorso-basal edge: [0] dark, concolorous with base; [1] with large, elongate whitish mark.
2	60		Mesopleuron centro-anteriorly: [0] without or at most with inconspicuous swirling rugulosities; [1] with strong swirling rugosities, often stemming from crenulation associated with epicnemial carina.
3	40		Speculum: [0] at least anterior half, sometimes fully, polished smooth; [1] fully punctuate, matte.
4	61		Sternaulus: [0] delicate, faintly crenulate; [1] moderately deep, distinctly crenulate; [2] deep, strongly crenulate.
5	12	✓+	Subalar ridge: [0] mostly or fully dark; [1] yellowish or whitish.
6	90	✓+	Fore coxa: [0] white; [1] dark orange, even if with darkened areas; [2] black.
7	100	✓	Fore leg (except coxa): [0] mostly light colored; [1] mostly or fully in mid tones (i.e., neither dark nor light); [2] mostly or fully dark or in dark tones.
8	38	✓+	Mid coxa: [0] black; [1] dark with orange tinge; [2] reddish brown; [3] dark orange; [4] orange; [5] whitish.
9	93	✓	Mid leg (except coxa): [0] mostly light colored; [1] mostly or fully in mid tones (i.e., neither dark nor light); [2] mostly or fully dark or in dark tones.
10	41	✓+	Hind coxa: [0] dark with reddish tinge; [1] dark with orange tinge; [2] dark orange; [3] reddish brown; [4] orange red.
11	76	✓	Fore wing: [0] hyaline; [1] weakly to moderately infuscate.
12	50	✓	Fore wing spots: [0] 1–2 even if weak; [1] absent.
13	57		Fore wing crossvein 1 cu-a: [0] distinctly basad of 1M+Rs; [1] opposite or approximately opposite of 1M+Rs.
14	11		Ramulus: [0] absent (vein smooth); [1] a small stub or absent but position indicated by venation bent where 1m-cu meets 1-Rs+M; [2] very long.
15	50		Propodeum sculpturing: [0] rough, much stronger than on metapleuron; [1] delicate, nearly as fine as on metapleuron; [2] very delicate, anterior transverse carina laterally vanishing.
16	32		Propodeum, V- or Y-shaped carinae arising from ATC towards propodeal sulcus: [0] absent; [1] present but inconspicuous; [2] delicate but fully developed; [3] delicate and quite short; [4] moderately strong; [5] strong, even if faint near anterior margin of propodeum.
17	53		Propodeum, short mid-longitudinal carina: [0] absent; [1] present.
18	31	✓	S1: [0] dark with reddish; [1] goldenrod; [2] reddish brown.
19	33	✓+	T1 apex: [0] with small to large whitish or yellowish spot; [1] concolorous with dark surroundings, or a little lighter.
20	U	✓+	T2 apical margin: [0] with uniformly narrow golden stripe, even if faint in darker; [1] diffusely reddish brown; [2] with wide whitish stripe.
21	71	✓+	T5: [0] meso-apically with conspicuous whitish; [1] uniformly colored.
22	90	✓+	T6: [0] mesally conspicuously whitish; [1] uniformly colored.
23	86	✓+	T7: [0] mesally conspicuously whitish; [1] mesally with very small whitish spot; [2] uniformly colored.

*C*, color pattern features, also coded with RGB where marked with a (+) plus sign; *N*, number of the character in the data matrix; *Ri*, retention index (acc. Fig 10A); *U*, uninformative.

To associate the putative males of *C*. *metamorphus* **sp**. **nov**. with the respective females, in light of an unusually high degree of sexual dimorphism for Cryptini, we sequenced a fragment of the mitochondrial *gene cytochrome oxidase I* using primers for the “barcoding” region (using LCO and HCO primers from Folmer et al. [20]) for one female (FAS333) and one male (FAS2144) collected in the same area (BRAZIL, ES, Cariacica, Duas Bocas, Alto Alegre, Primary Forest, 501 m, 20°16'54.4"S, 40°31'20.7"W) (female on 05-20.X.2016, male on 10-26.IV.2017). DNA extraction, amplification and sequencing were performed using the same protocols as in Scherrer [21]. The two sequences were aligned using the Geneious alignment algorithm (Geneious Prime v.2019.0.4, Biomatters), and compared in their pairwise distance to check for specific association.

### Color data

Extraction of color features was performed according to an original procedure, as follows. All female specimens were photographed laterally (head + mesosoma) and dorsally (metasoma), with focus stacking (see Brecko et al. [22]). RGB values from uncompressed, lossless (TIFF) images (S1 File) were directly used as color data. Although RGB values are device-dependent (e.g. [23]), any given camera will produce highly comparable results, representing a much more precise proxy for true colors than descriptive or discrete coding based solely on the human eye. Most photographs (84%) used in this procedure were shot with the same equipment; the remaining images were obtained from two other cameras, but their colors were calibrated according to the method developed by Reinhard et al. [24], after adjusted for illuminance, which also interferes with RGB values.

To mitigate the illuminance problem, one of the images was selected as a standard, its background manually removed (i.e., erased to white), and the normalized average exposure of the image of the specimen itself was calculated as

$$\frac{\sum_{i=n}^{n}\left(R_{i}+G_{i}+B_{i}\right)}{3n255}$$

The exposure of the specimens depicted on each of the remaining images was then heuristically adjusted, using Bézier curves, to match as precisely as possible that of the standard. In this process, all specimens, except the standard, were roughly isolated from the background through a simplified algorithm–exclusion of the top 55% brightest pixels. The resulting images ended with nearly identical average exposure for the specimens, with an average difference of only 0.05%.

Color samples were then extracted from the images from 26 standardized points on each specimen (Table 2). It is noteworthy that the key objective of each sampled point was to get a representative color for a given *area* of the body, such as the lateral portion of the gena, the lateral side of front coxa, etc. Therefore, it is not necessary that the sampled points correspond to precise anatomical equivalents for each specimen, i.e., morphometric landmarks.

**Table 2:** External body areas sampled for RGB values on all available female specimens of *Cryptoxenodon metamorphus* sp. nov. Specimens were photographed in lateral (left) view for characters 0–12, and dorsal view for the remaining characters.

Num	Tagma	Where	Sampling objective
1	Hd	Gena, lateral subventral	Main color
2	Ms	Pronotum, latero-central area	Main color
3	Ms^+^	Mesonotum, left lateral lobe	Main color
4	Ms^+^	Mesopleuron, subalar ridge	Lightest point
5	Ms	Mesopleuron	Main color
6, 7	Ms^+^	Fore coxa external face	Two most representative colors
8, 9	Ms^+^	Mid coxa external face	Two most representative colors
10, 11	Ms^+^	Hind coxa external face	Two most representative colors
12	Ms	Metapleuron	Main color
13	Ms	Apex of propodeal apophysis	Lightest color
14	Ms	Petiole base, ventro-lateral	Most reddish area
15	Mt^+^	T1, apical area	Most representative color of apical margin
16, 17	Mt	T2, main area	Two most representative colors
18	Mt^+^	T2, apical margin	Most representative color of apical margin
19, 20	Mt	T3, main area	Two most representative colors
21	Mt	T3, apical margin	Most representative color of apical margin
22, 23	Mt	T4, main area	Two most representative colors, apical margin excluded
24	Mt^+^	T5	Color at the center of the white spot, if present, ***or*** Most representative color of tergite, apical margin excluded
25	Mt^+^	T6	Same as above
26	Mt	T7	Same as above
27	Mt^x^	T8	Same as above

*Hd*, head; *Ms*, mesosoma; *Mt*, metasoma; *Num*, sample point number; (+), sampling points with equivalent coded characters in the morphological matrix.

To investigate the darkening of specimens (Fig 10), the *perceived* brightness of each color sample was calculated according to Finley [25], as $\sqrt{0.2989R^{2}+0.5870G^{2}+0.1140B^{2}}$ for linear 0–1 RGB values. This value ranges from 0.0 for entirely dark to 1.0 for entirely white.

The selected sampling points on each image were first registered as image coordinates, using the tpsDig software [26]. Actual RGB values were then extracted from pixels around each sampling point, within a radius of 2 pixels, thus equivalent to an area of 13 pixels. The representative RGB values used for each sample was the average value of each set of Rs, Gs and Bs from the corresponding set of 13 pixels. The resulting matrix, of 50 specimens × 78 variables, has 174 missing values, due to damaged or missing parts, and 3726 informative cells (S1 File). Altitude and latitude data were then superimposed on the resulting PCA, on a 3D chart (Fig 9). Exposure adjustments, RGB sampling, and graphics, were all performed or built with Python scripts written for these specific aims.

Since some of the RGB sample points were directly equivalent to color characters coded for the morphological matrix (Tables 1 vs. 2; characters and body areas marked with a plus sign), an alternative character matrix was assembled by replacing all 9 characters marked on Table 1 with data from the 12 equivalent RGB points indicated in Table 2 (that is, 36 RGB characters), as well as by adding all of the remaining 15 RGB points (45 characters) (S2 File); the aim of this matrix was to produce a comparative interpretation (Fig 6).

### Intraspecific variation

To investigate for the existence of a latitudinal vs. altitudinal cline in morphological variation, a Multiple Factor Analysis (MFA [27, 28]) of the morphological variation matrix (categorical data), including RGB values (continuous data) was performed. An MFA is more accurate than Principal Component Analyses (PCA) when categorical and numeric data are to be analyzed together. The MFA was performed with the R package PCAmixdata [29].

In order to evaluate the degree of association of altitude and latitude with the observed morphology, each of these two variables were compared with the categorical (Table 4) and the continuous (S1 File) characters. The available latitude data, however, is clumped into four distinct subgroups (Fig 5A), preventing its investigation as a continuous variable with correlation indexes (see Aggarwal & Ranganathan [30]). Because of that, latitude data was investigated only as a categorical, ordered variable, with four states: [0] 0 to 5˚N, [1] 0 to -5˚S, [2] -10 to -15˚S, [3] < -15˚S. The correlation between continuous vs. categorical variables was calculated with point-biserial correlation (Sheskin [31], here implemented in Python with the library scipy.stats.pointbiserialr) for binary characters, and with polyserial correlation [32, 33] (implemented in R with the package polycor, with ML = TRUE) for characters with three or more states. For assessing the correlation between continuous variables, such as altitude vs. RGBs, the Pearson’s product moment correlation was calculated; all prerequisite conditions listed by Aggarwal & Ranganathan [30] were graphically checked in all cases; where doubtful, linearity was further verified by comparing multiple curve fits with the Akaike information criterion [34].

Correlation between sets of categorical variables was measured using the Cramér’s V index [35], calculated using a custom Python script extracted from the dython library, by Zychlinski [36]. Since all mentioned indexes are somewhat related (they are all conceptually derived from the Pearson’s index) and have equivalent ranges for their absolute values (0.0 to 1.0), comparative plots were presented combining different correlation indexes (Fig 7B–7C).

A separate PCA for color features was performed, using data from nearly all available female specimens, according to the procedures described in the item Color data, above. Missing values were handled through iterative imputation [37].

### Ecological niche modelling

The potential distribution of the species was modeled from all known occurrence records, using climatic data obtained from the Inpe database (www.dpi.inpe.br), which includes all Worldclim variables and others, with 57 variables total, each one represented by monthly data from 1950 to 2000. Modeling was performed using MaxEnt [38], with 30% random test percentage, 30 replications, the standard convergence limit (10–5), maximum number of iterations (5000), and other standard resources. From these results, we further analyzed the top 15 variables which contributed most to the results (as measured by MaxEnt) with a PCA, to measure the degree of correlation between them. Pairs of variables were compared according to the highest values for PC1; if correlation for a given pair was larger than 0.7, the contribution of each variable was recalculated with jackknifing, and that with the lowest contribution was discarded. This was repeated until only 4 variables remained–this number follows a general rule of thumb that sample size (number of unique occurrences; 43 herein) should be 10 times larger than the number of predictors used for modelling [39]. To these, we also added the variable altitude, because of its relevance in this study. PCA and correlation of variables were performed with the R packages *raster*, *rgdal* and *vegan*. The following five variables, with a resolution of 2.5 minutes of arc, were used: alt (altitude), bio3 (isotermality, obtained as bio2/bio7*100), decl (declivity), prec9 (total precipitation of August), and prec12 (total precipitation of December). In order to evaluate distinct ecological scenarios, we calculated two different occurrence thresholds, one considered to be very conservative (Maximum Training Sensitivity Plus Specific) and one more inclusive (Minimum Training Presence).

## Results

### Phylogeny

Seven molecular loci were sequenced (Fig 2). In addition, the character-coding for the external morphology of *Cryptoxenodon* **gen**. **nov**. is shown in Table 3.

**Table 3:** Character-coding for *Cryptoxenodon metamorphus* sp. nov.

Format	Coding
0	02002002a1 0010-a0000 001b0b1100 0000a12000 00000a02b1 101002-1c3 aa11011001 2121010100 01110101aa 200a011000 0a0000010
1	13113113?d 21121-d111 1112e1e221 11111d2311 111111d13e 2212113-2f 3dd2212211 2323212121 112221212d d311d12211 11d1111121

For convenience, it is presented in two formats: (0) compatible with the character-set, and (1) compatible with the data matrix of Santos [12] (see explanation in Material & Methods). Multistate for Format 0: a = [01]; b = [12], c = [14]. Multistate for Format 1: d = [12], e = [23], f = [25].

**Fig 3:**
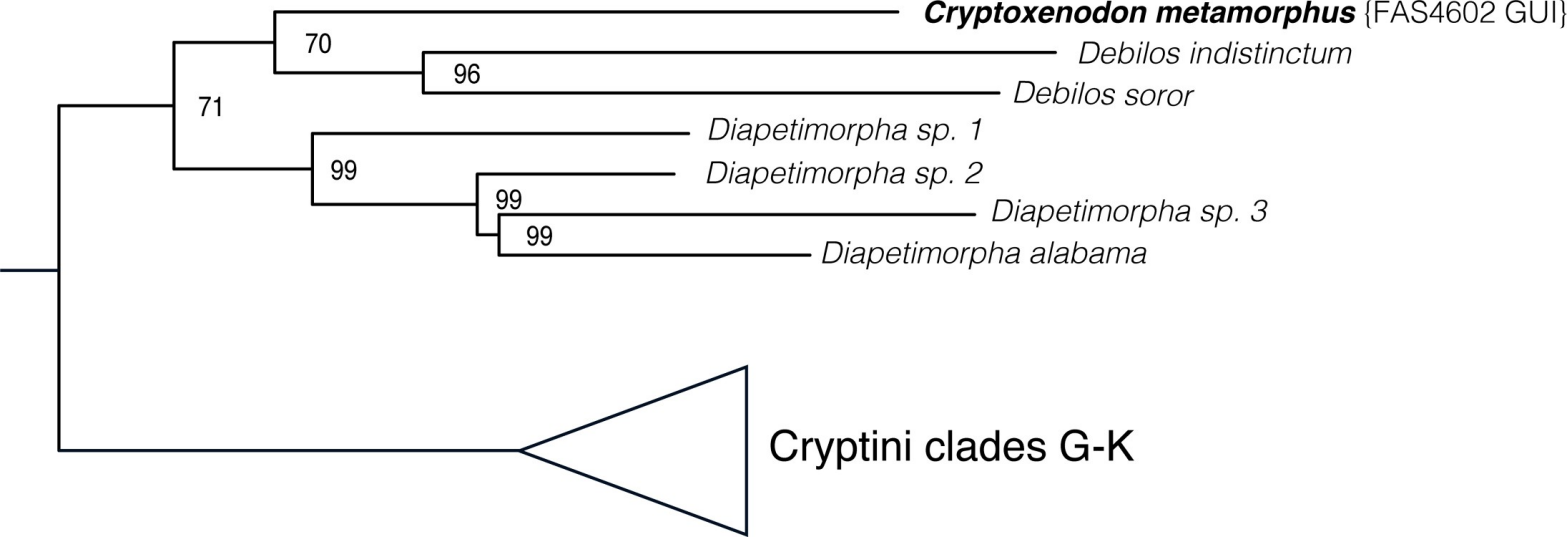
Phylogenetic placement of *Cryptoxenodon* gen. nov. in the maximum-likelihood tree using the molecular and morphological matrix of Santos [12]. Clade names refer to the major genus-groups identified in this previous work. See S3 File for the complete tree.

Maximum-likelihood analyses of the combined molecular and morphological dataset place *Cryptoxenodon* **gen**. **nov**. as sister to *Debilos* Townes, with moderate support (bootstrap = 70) (Fig 3). The clade *Cryptoxenodon*+*Debilos* was sister to *Diapetimorpha* Viereck, again with moderate support (bootstrap = 71). Both *Debilos* and *Diapetimorpha* individually are strongly supported (bootstrap = 96–99). The overall tree topology (S3 File) is remarkably like the one recovered by Santos [12] using different phylogenetic inference software (GARLI 2.0 [41]).

The results strongly support the classification of the new taxon as a separate, independent genus, phylogenetically distinct and morphologically diagnosable from both *Debilos* and *Diapetimorpha*, as well as from all other Neotropical taxa.

### Species delimitation

**Fig 4:**
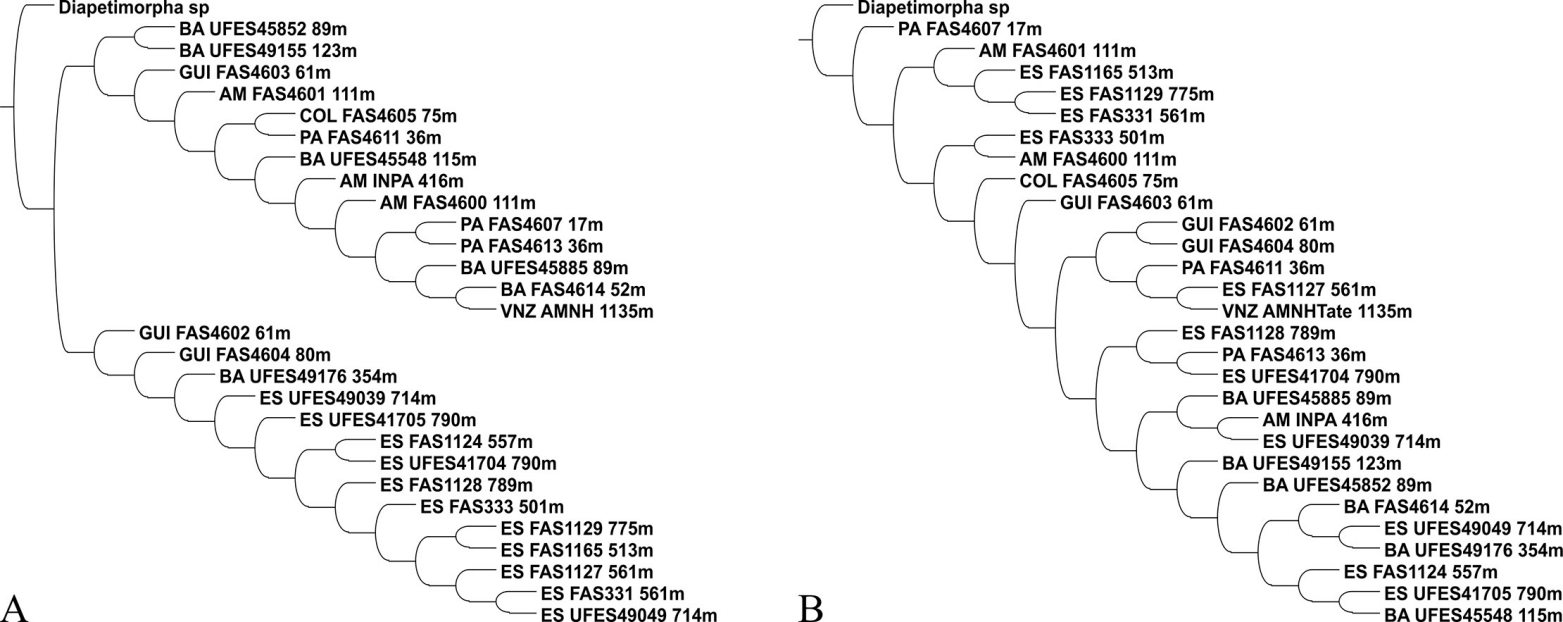
Most parsimonious interpretations for the observed morphological variation of *C*. *metamorphus* sp. nov. (females). (A) Using discrete characters only (Tables 3 and 4). K = 4.765625, Fit = 8.31342, Rearrangements tried: 1,052,652,173. Length = 164, CI = 26, RI = 57. (B) Combining discrete characters with RGB as quantitative characters. K = 20.048829, Fit = 27.68569, Rearrangements Tried: 996,214,326. If this topology is used with the discrete character matrix, Length = 275, CI = 16, RI = 17. GUI corresponds to French Guiana, COL to Colombia, and VNZ to Venezuela; two-letter tags correspond to Brazilian states. Both analyses recovered a single tree found with implied weights, with the constant of concavity (K) defined by the algorithm setk.run.

Morphological variation among the examined specimens for the new genus proved to be extensive, and occurrence records are clustered in two apparently disjunct population groups. The obvious hypothesis to be tested was that two or more species were present among the examined material. The strict consensus of the most parsimonious tree for the coded characters (Table 4) recovered clades containing representatives of widely separated populations, such as ES + BA + GUI (Fig 4A), or no convincing clades at all (Fig 4B). A PCA analysis of the mixed character matrix (Fig 6) also did not recover discrete groups. These results indicate that there is no reasonable support for splitting, pointing instead to a single, even if variable species.

**Table 4:** Data matrix used in the analyses of morphological variation. Coding reflects all variation observed for all available females of *Cryptoxenodon metamorphus* **sp**. **nov**. First specimen (FAS579*) is a *Diapetimorpha* sp., used as outgroup; other specimens ordered from the lowest to the highest altitude. *Alt*, altitude, in meters; *Coden*, State abbreviations (Brazil) or coden for the country.

Database	Country	Coden	Alt	Latitude	Character number
					11111111112222 012345678901234567890123
FAS579*	Brazil	ES	513	20º17'02.6"S	000010015130000030202000
FAS4607	Brazil	PA	17	01°44'12.8"S	100010100100010000000110
FAS4611	Brazil	PA	36	01°44'14.0"S	000110100000110140100110
FAS4613	Brazil	PA	36	01°44'14.0"S	100111100000010020000110
FAS4614	Brazil	BA	52	14°46'00.0"S	100020103020010050010000
FAS4602	French Guiana	GUI	61	04°43'22.22"N	000020110100011050010002
FAS4603	French Guiana	GUI	61	04°43'22.22"N	000121113100001230210010
FAS4605	Colombia	COL	75	01°55'00.0"S	000111100020102151110110
FAS4604	French Guiana	GUI	80	04°02'00.0"N	001020110100101051010001
UFES45852	Brazil	BA	89	14°20'48.0"S	000010210100000041010010
UFES45885	Brazil	BA	89	14°42'69.0"S	100010100000010021010010
FAS4600	Brazil	AM	111	02°57'48.3"S	100011100000101041010110
FAS4601	Brazil	AM	111	02°57'48.3"S	010100100101001110010010
UFES45548	Brazil	BA	115	14°34'30.0"S	001011101011000050111110
UFES49155	Brazil	BA	123	14°56'49.0"S	001011210100010050110110
UFES49176	Brazil	BA	354	14°59'51.0"S	001021110100000001110002
INPA	Brazil	AM	416	03°44'45.0"S	000021103020001040110110
FAS333	Brazil	ES	501	20°16'54.4"S	000021212201000050110002
FAS1165	Brazil	ES	513	20°17'02.6"S	000011220221000051110002
FAS1124	Brazil	ES	557	20°17'19.9"S	001021213221000041110002
FAS331	Brazil	ES	561	20°17'26.4"S	001021224241100051210002
FAS1127	Brazil	ES	561	20°17'26.4"S	001021220201101051110002
UFES49039	Brazil	ES	714	20°27'53.0"S	001021110101000051010002
UFES49049	Brazil	ES	714	20°27'53.0"S	001021222231100051010002
FAS1129	Brazil	ES	775	19°55'16.4"S	000021220221000051110001
FAS1128	Brazil	ES	789	19°53'41.0"S	000021212231000051210002
UFES41704	Brazil	ES	790	20°02'31.1"S	001021213221000041210002
UFES41705	Brazil	ES	790	20°02'31.1"S	001021112131000050210001
AMNH156	Venezuela	VNZ	1135	03°30'48.0"N	100101103041012150010000

Comparison of the COI sequences obtained for male and female specimens from the same locality (20°16'54.4"S, 40°31'20.7"W) revealed 100% sequence identity (GenBank Accession numbers MT085820 and MT085821), confirming the association made based on morphology in spite of considerable sexual dimorphism (see section Taxonomy, below). Compared to the specimen from French Guyana used in the phylogenetic analyses, there was 5.7% of pairwise divergence.

### Altitudinal and latitudinal clines

**Fig 5:**
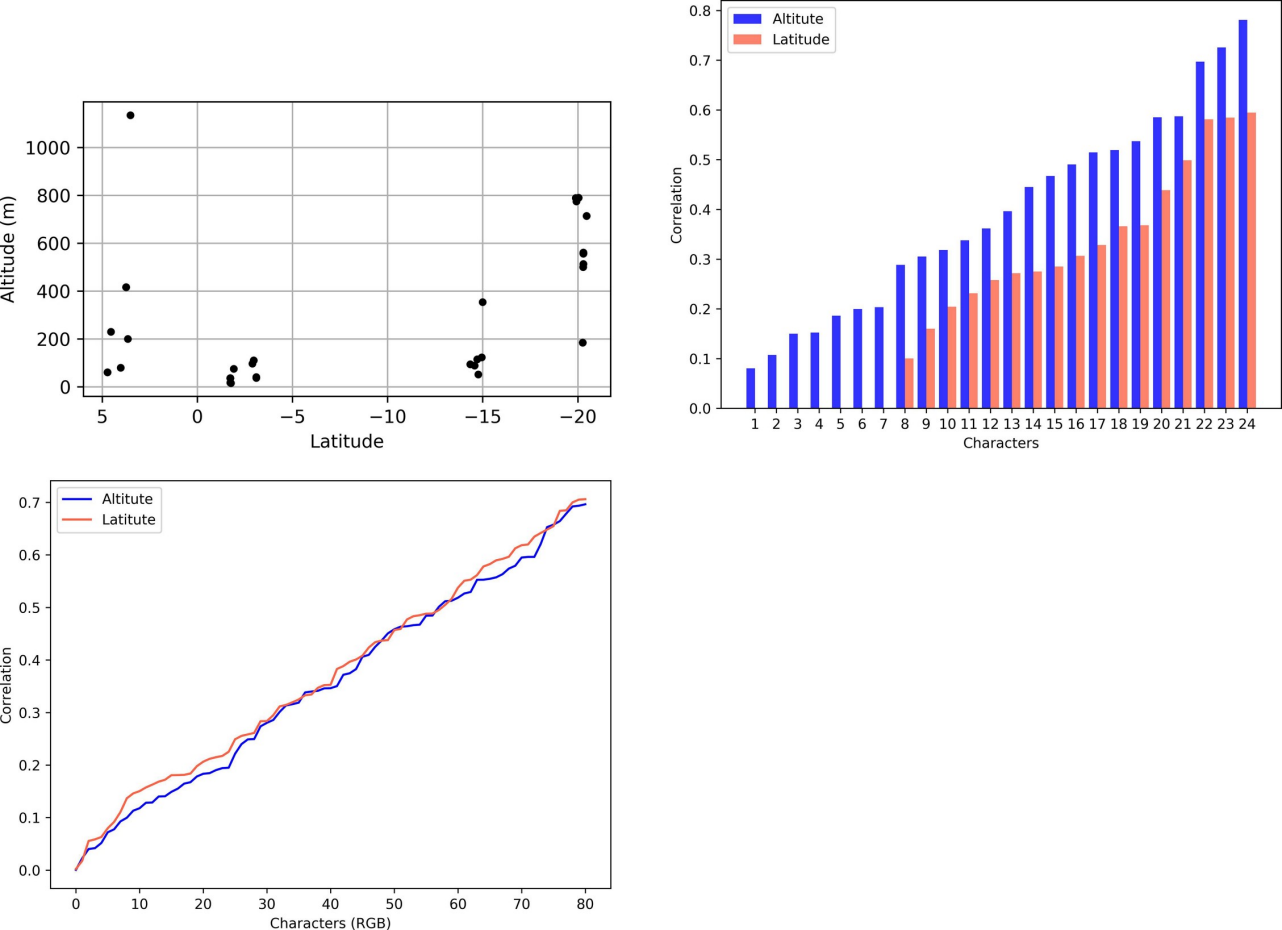
Relationship between altitude and latitude, and their correlation with all discrete and continuous characters investigated for female specimens of *C*. *metamorphus* sp. nov. (A) Latitude vs. Altitude data for all available females. (B) Sorted absolute values of point-biserial and polyserial correlation for altitude (blue, 1^st^ series), and of Cramér’s V index for latitude (red, 2^nd^ series) for 24 categorical morphological characters (those of Table 3, but freely sorted here). The difference between the average correlation values of each series is 0.149367. (C) Absolute values of Pearson correlation for altitude (blue, bottom curve) and polyserial correlation for latitude (red, on top) vs. 81 RGB (color) characters. The difference between the average correlation values of each series is only 0.014824 in favor of latitude. Characters on charts B and C were independently sorted for the altitude and the latitude series, to highlight the net differences in correlation.

The available data for latitude and altitude data were not linearly related, with latitude data showing distinct subgroups (Fig 5A). This prevents the usage of a correlation coefficient (see [30]), making it difficult to state precisely how much latitude is independent from altitude in this particular study.

The categorical morphological variables (Table 4) correlated consistently higher with altitude than with latitude (average correlation with altitude = 0.393207; with latitude = 0.243840; average difference = 0.149367) (Fig 5B), but results considering exclusively the color features (continuous RGBs) do not distinctly favor one variable over the other, with only 0.014824 average difference favoring latitude (average correlation with altitude = 0.356327; with latitude = 0.371151) (Fig 5C). In all cases there is a wide range of correlation values, ranging from nearly 0.0 to about 0.8.

**Fig 6:**
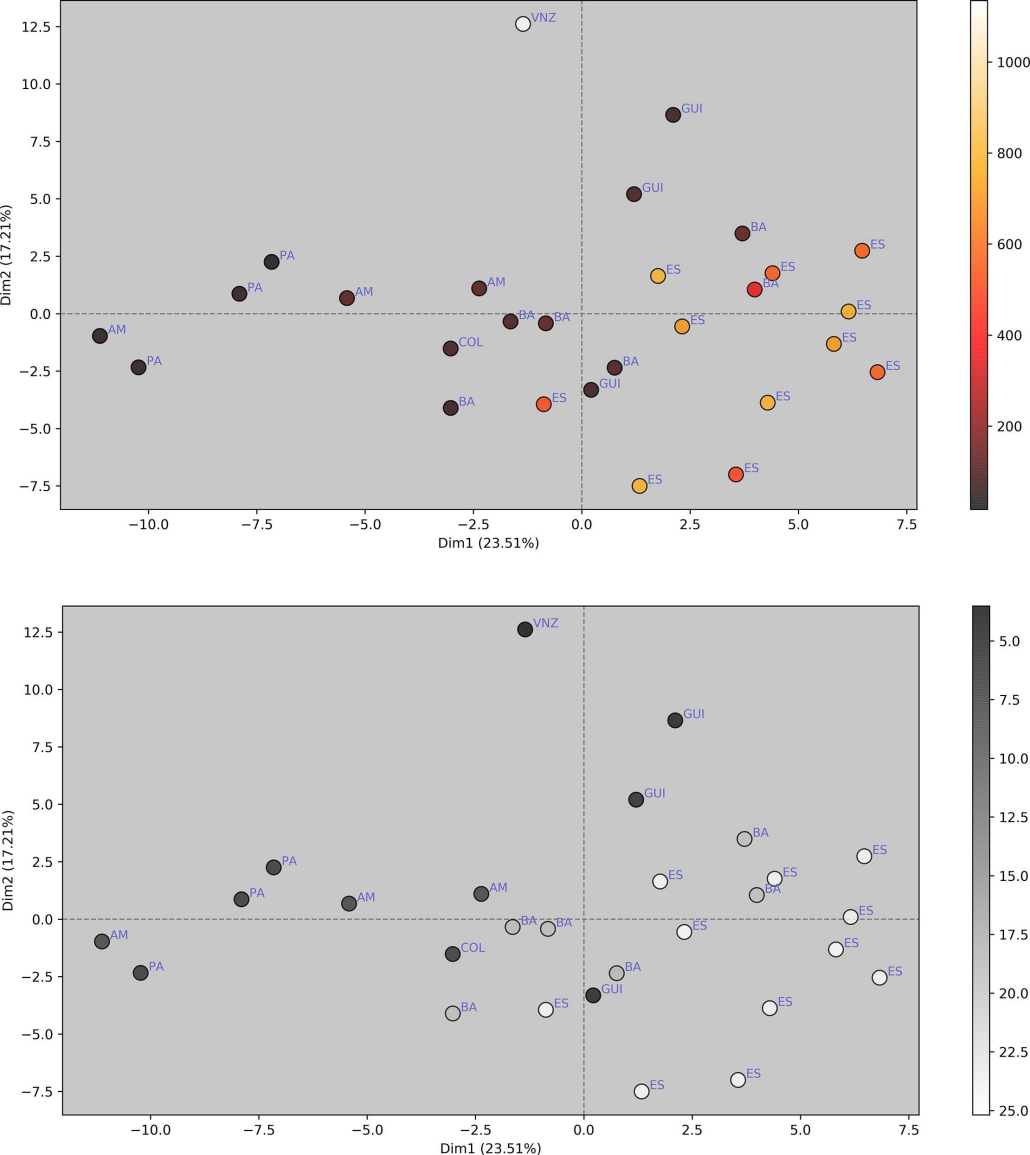
Multiple Factor Analysis (PCA of mixed data) grouping all female specimens of *C*. *metamorphus* sp. nov. according to discrete (morphology) and continuous (RGB values) characters (Tables 2 and 4). (A) Color-coded according to altitude. (B) Color-coded according to latitude. Locality abbreviations as in Table 4.

**Fig 7:**
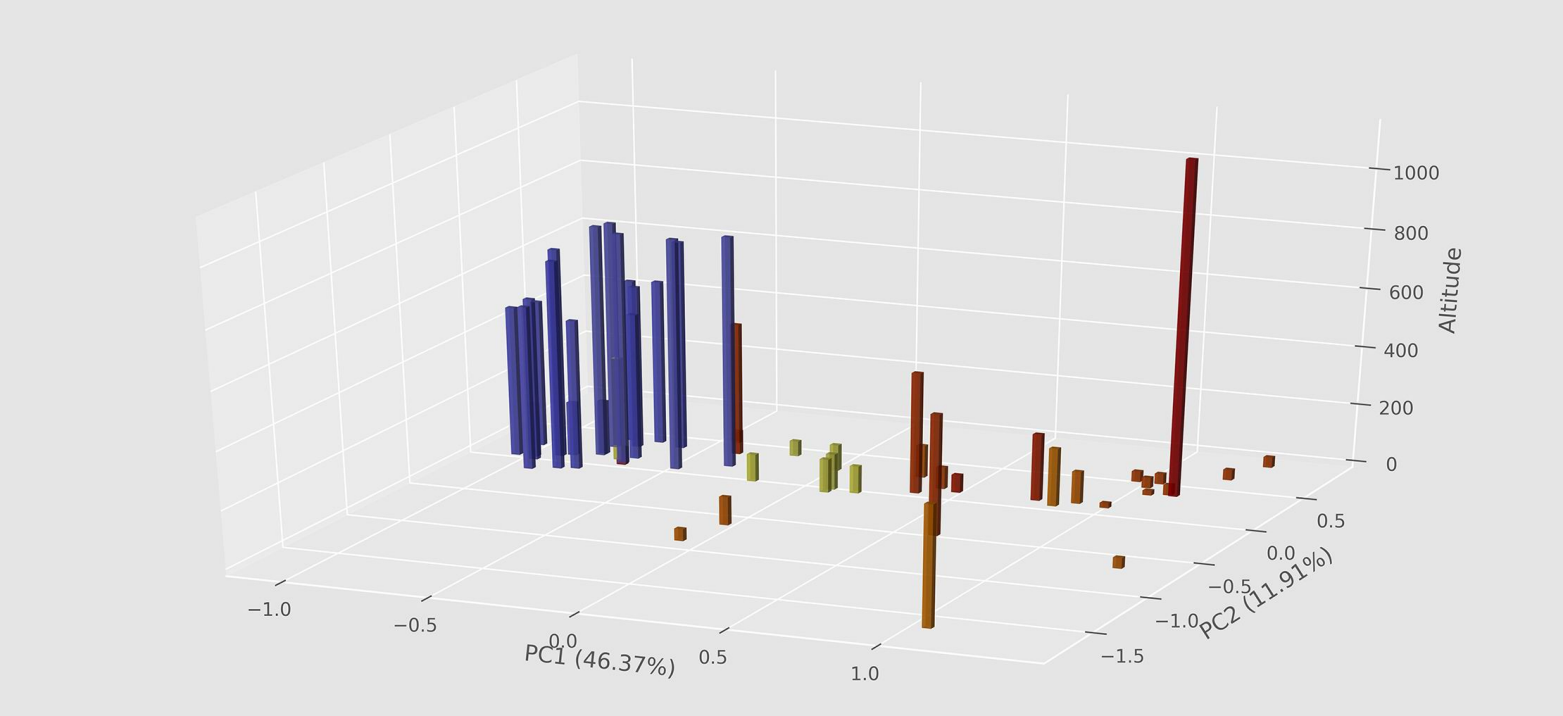
PCA of 27 RGB values (= 81 variables) extracted from all available females (51 specimens) of *C*. *metamorphus* sp. nov., plotted against altitude (size of bars) and latitude (color of bars, from blue on southeast to red on north).

**Fig 8:**
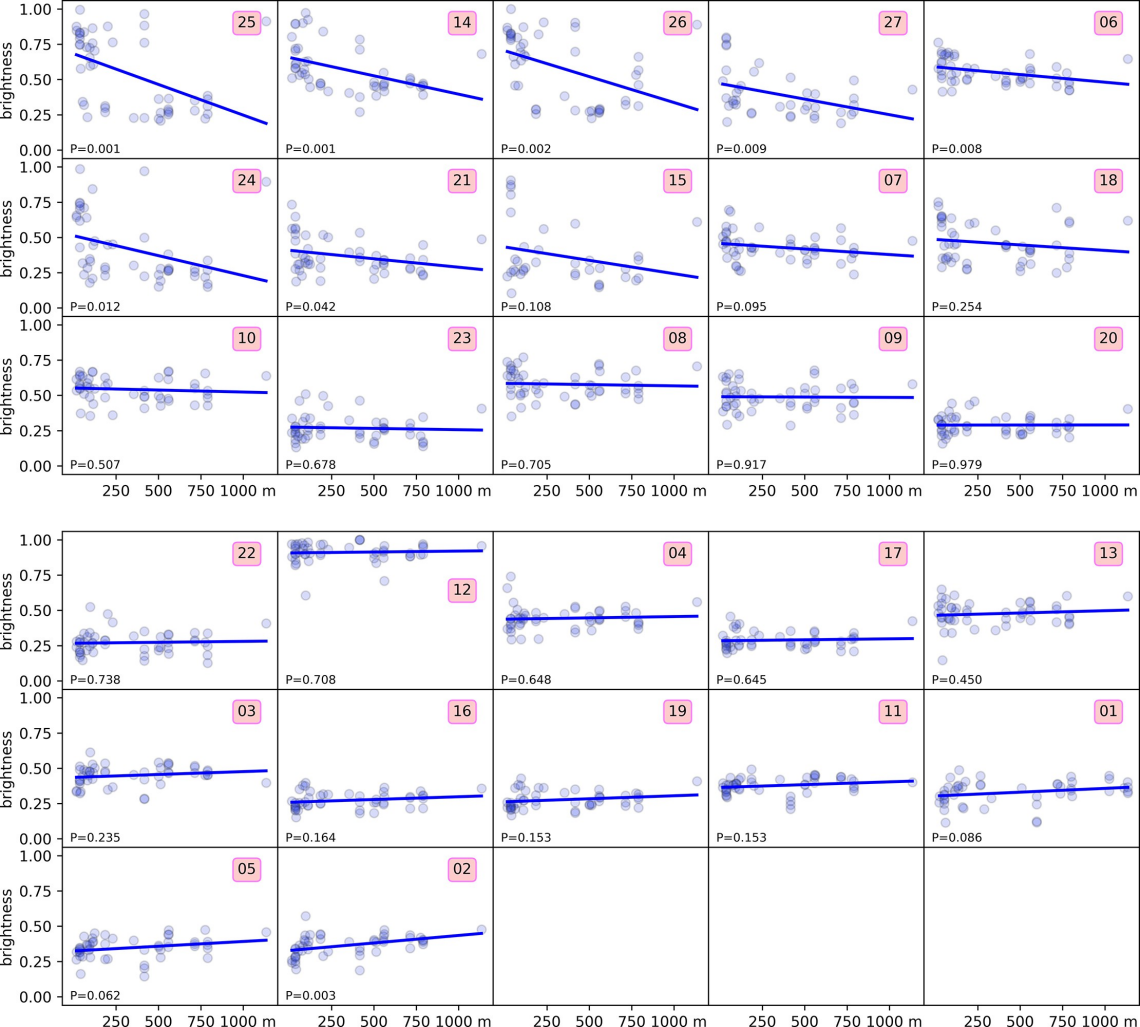
Relationship between brightness with altitude for each of 27 external body areas sampled for RGB values on all available females of *Cryptoxenodon metamorphus* sp. nov. (51 specimens). The number on each box inside the subplots corresponds to the sampled body area, acc. Table 1. Subplots ordered from the most decreasing to the most ascending curve (linear best fit). The respective P value is shown in each subplot.

The MFA analysis (Fig 6) shows some stratification for specimens from both different altitudes (Fig 6A) and latitudes (Fig 6B), albeit this is evident only for the first axis (Dim1). This is perhaps most evident in the plot with altitude + latitude data overlaid on the PCA results for RGB data from all females (Fig 7). Darkening in at least 26% (7/27) of the investigated body areas, however, increases significantly with altitude (P < 0.05) (Fig 8, first seven subplots), but only in one case it shows a clear (P = 0.003) but very weak opposite response (last subplot).

These results seem therefore consistent in supporting a dual cline, with populations being concurrently affected by both altitude and latitude, albeit possibly a little more intensely affected by altitude, as suggested by the differences in Fig 5B.

## Discussion

### Phylogeny

Except by the exceptionally long mandibles, *C*. *metamorphus* **sp**. **nov**. is externally remarkably similar to species of *Diapetimorpha*. However, the combined morphological and molecular phylogenetics analysis was unequivocal that the new taxon falls out of the *Diapetimorpha* clade, which was represented by four species. In fact, *C*. *metamorphus* **sp**. **nov**. was shown to be most closely related to *Debilos*, providing further support for its treatment as a distinct genus.

In the analyses of Santos [12], the position of the clade *Diapetimorpha*+*Debilos* was variable within the Cryptini tree, in some analyses recovered as sister to the *Lymeon* group (and therefore arguably part of it), in others as sister to a lineage containing three major clades (*Osprynchotus* group + (*Trachysphyrus* group + *Agrothereutes* group)). Herein a third result was recovered, with the clade (*Diapetimorpha* + (*Cryptoxenodon* + *Debilos*)) recovered as sister to an even broader clade (S3 File). This instability, combined with the relatively low bootstrap values at higher levels of the phylogeny, highlights the challenge in reconstructing higher-level cryptine phylogeny even after sequencing multiple genes. In phylogenomic analyses using ultraconserved elements [42], the clade (*Diapetimorpha* + *Debilos*) was recovered as sister to the *Lymeon* group, but those analyses had much smaller taxon sampling (70 cryptine species).

### Species delimitation

The combination of multiple sources of evidence and analytical frameworks did not support the recognition of more than one species of *Cryptoxenodon* **gen**. **nov**. Although the specimens examined display a considerable amount of morphological variation, no method could point out to a discrete division among the observed populations. The pairwise genetic distance between the sequenced specimens, from French Guyana and southeastern Brazil (5.7%), was higher than the 3% threshold often used to separate species by DNA barcoding initiatives (e.g. [43]), but high levels of intraspecific divergence for COI sequences are not uncommon among insects, including studies recording 30.8% for cockroaches [44], 31.15% for thrips [45], 21.8% for mosquitoes [46], 18.3% for praying mantises [47], and 17.5% for *Drosophila* [48]. Sequencing of only two specimens does not allow for further inferences of population structure in *C*. *metamorphus*, but further sequencing efforts were not part of the original aim of this work, and would not be possible for most specimens due to previous processing with ethyl acetate, that destroys DNA [49].

Compared to other cryptine genera, *C*. *metamorphus* **sp**. **nov**. shows an unusual level of sexual dimorphism (see Taxonomy section for a detailed account), complicating female-male associations. The 100% identity observed in COI sequences represents unequivocal corroboration of the morphology-based association and will help in future morphological interpretation of similar character systems in other variable species.

### Morphological clines

Results presented above make a strong case for a dual cline, altitudinal and latitudinal, with a slightly more intense influence of altitude. The underlying reasons for this, however, are not obvious. Water loss, temperature, and UV light, for example, might be involved.

#### Altitude

Higher altitudes are not necessarily directly linked to reduced humidity, but elevated areas, such as hills or cliffs within a forest, can sometimes be drier than the plateau (e.g. [50]). This is significant because ichneumonids are especially vulnerable to desiccation [51], and need to drink water daily [51, 52]. Shapiro and Pickering [53] also noted that parasitoids of larval Lepidoptera, such as many Ichneumonidae, may be further affected by desiccation by having to search for hosts up on the foliage, where it is dryer than near the soil.

It has been suggested that increased melanization may reduce cuticular permeability and allow insects to better resist desiccation [54], and such an effect was shown to appear rapidly from selection in laboratory experiments [55–58], suggesting that melanism and desiccation resistance are linked through differences in cuticular permeability [54].

Temperature could also play a role. Abe et al. [59], for example, elegantly demonstrated that colder temperatures produce darkened forms of *Meteorus pulchricornis* (Wesmael) (Braconidae). This is in line with the thermal melanism hypothesis [60, 61], which postulates that dark individuals are at an advantage under conditions of low temperature, *i*.*e*., higher altitudes or latitudes, because they heat up faster at a given level of solar radiation. This efficient use of radiation for heating allows an increased period of activity for dark individuals in cold environments, which may in turn increase success in feeding, escaping predators, and finding mates [60, 62], and even increasing fertility [63].

Dark coloration at higher altitudes also may relate with protection against UV radiation, which increases with altitude [64], and intense ultra-violet may be a contributing factor in the production of dense pigments [65], which serve to protect the deeper and delicate tissues from injury by this radiation.

While the melanism-desiccation hypothesis or the thermal melanism hypothesis seem inadequate to explain variations such as cuticular microsculpturing or wing venation features, they are both consistent with our data, which shows darker individuals of *C*. *metamorphus* **sp**. **nov**. in higher altitudes and colder areas. Similar altitude-related color variation is also reported for other insect groups, such as butterflies [60, 66] and grasshoppers [67, 68]. For *C*. *metamorphus* **sp**. **nov**., however, the average yearly temperature for the areas occupied by the northern populations is 24.8˚C, versus 21.0˚C for the southeastern populations, a much smaller variation (3.8˚C) than that investigated in previous studies, e.g. 15.0˚C in [59].

#### Latitude

Latitudinal clines, on their turn, are also reported for multiple aspects of the life of Hymenoptera, such as diversity of parasitoid bees and ichneumonids [69, 70], guild composition of parasitoid wasps [71], diapause in Braconidae [72], nest architecture in Vespidae [73], rates of predation in ants [74], and even dramatic variations in sex-ratio, for Pelecinidae [75–77]. Published investigations on the relationship between *morphological* traits and latitude, however, are usually limited to testing Bergmann’s rule, i.e., that size increases with latitude [78]. For the Neotropical region, it seems that only Oliveira et al. [79], working with leafhoppers (Cicadellidae), went somewhat further by showing that individuals from higher latitudes were not only larger and heavier, but also *darker* than those from lower latitudes.

Increasing latitudes generally imply lower temperatures, which are also characteristic of higher altitudes. This could suggest that the effect of latitude and altitude are similar, but temperature apparently is not a key factor for *C*. *metamorphus* **sp**. **nov**., as discussed above, and higher altitudes also affect a series of other variables to which insects are quite sensitive, such as humidity, intensity of UV light, atmospheric pressure, oxygen concentration, wind, and others.

### Taxonomy

***Cryptoxenodon* Supeleto, Santos & Aguiar, gen. nov.** (Figs 9–16)

**Fig 9:**
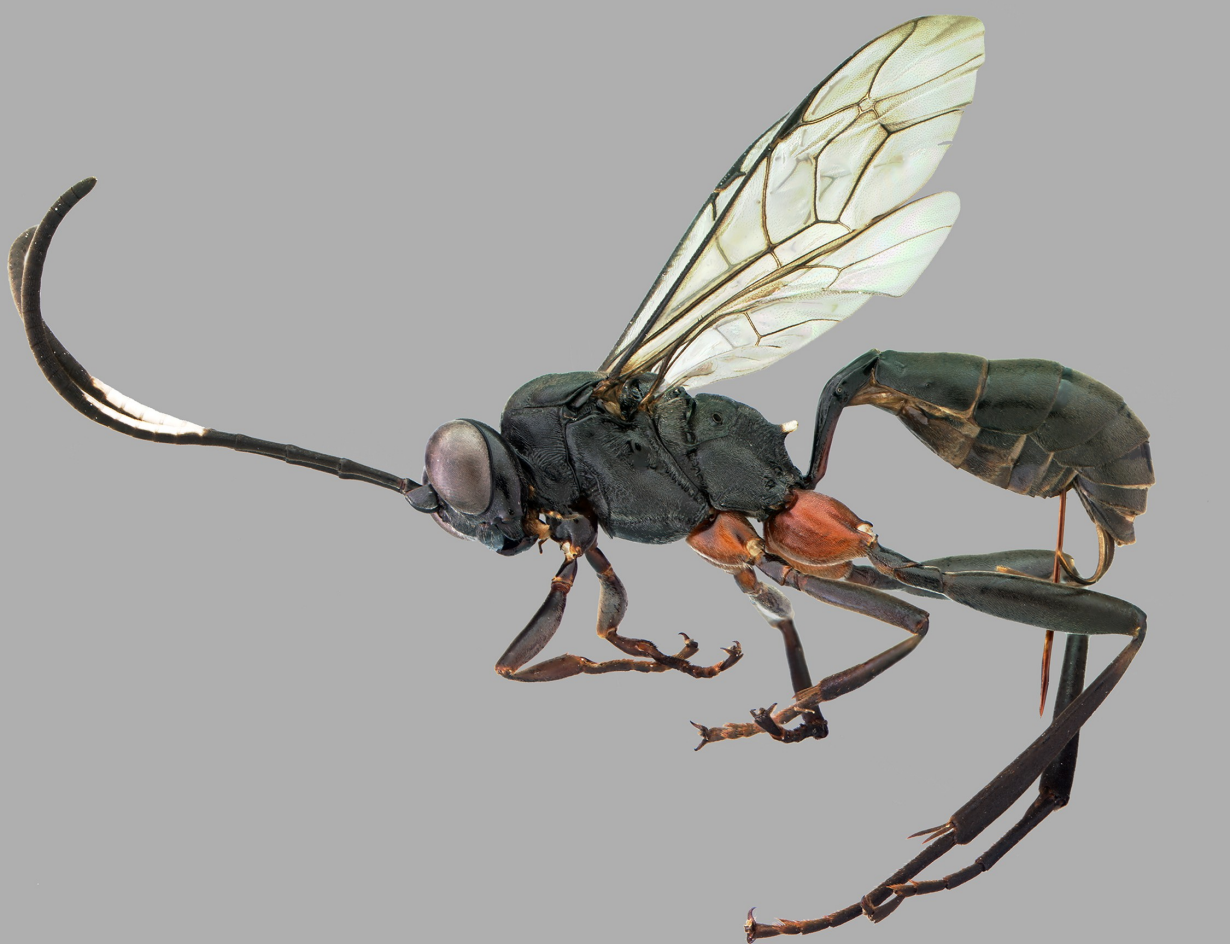
Holotype female of *Cryptoxenodon metamorphus* sp. nov. FAS1127, Alt. 561m, Brazil/ES. Habitus.

**Fig 10:**
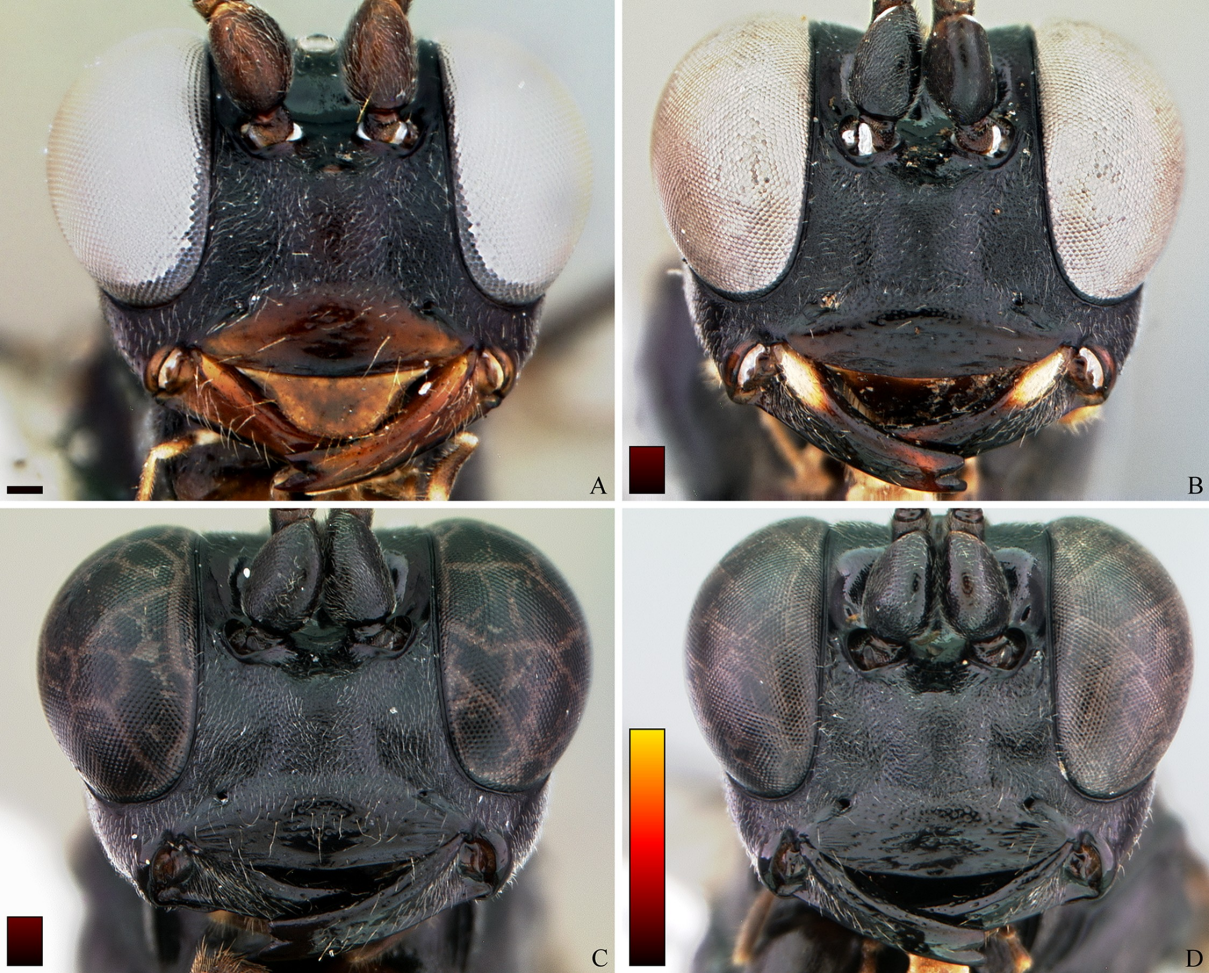
*Cryptoxenodon metamorphus* sp. nov. Female head in frontal view. (A) Paratype FAS4608, Alt. 16m, Brazil/PA. (B) Paratype FAS4601, Alt. 111m, Brazil/AM. (C) Paratype UFES45548, Alt. 115m, Brazil/BA. (D) Holotype FAS1127, Alt. 561m, Brazil/ES. Bars are proportional to the altitude.

**Fig 11:**
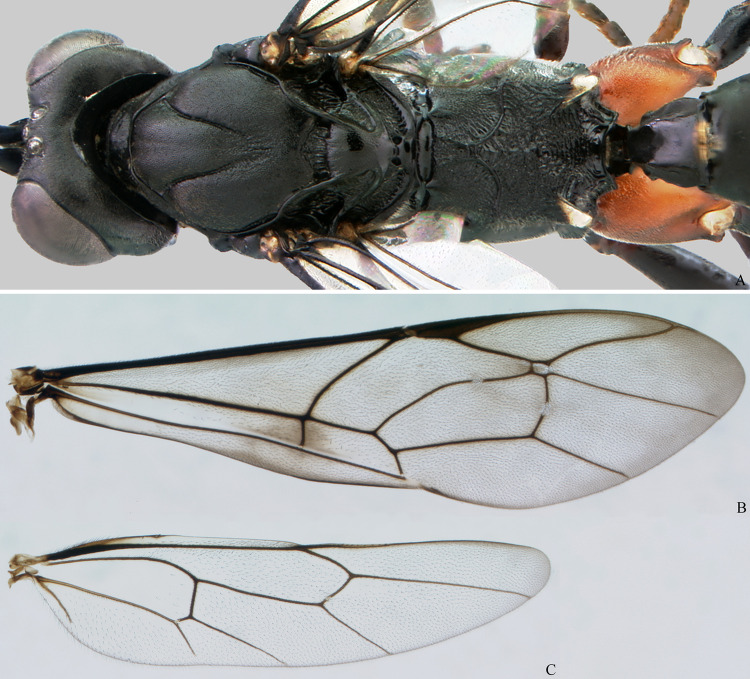
*Cryptoxenodon metamorphus* sp. nov. Female mesosoma and wings. (A) Mesosoma + first tergite; holotype FAS1127, Alt. 561m, Brazil/ES. (B-C) Fore and hind left wings, horizontally flipped; paratype FAS1131, Alt. 557m, Brazil/ES.

**Fig 12:**
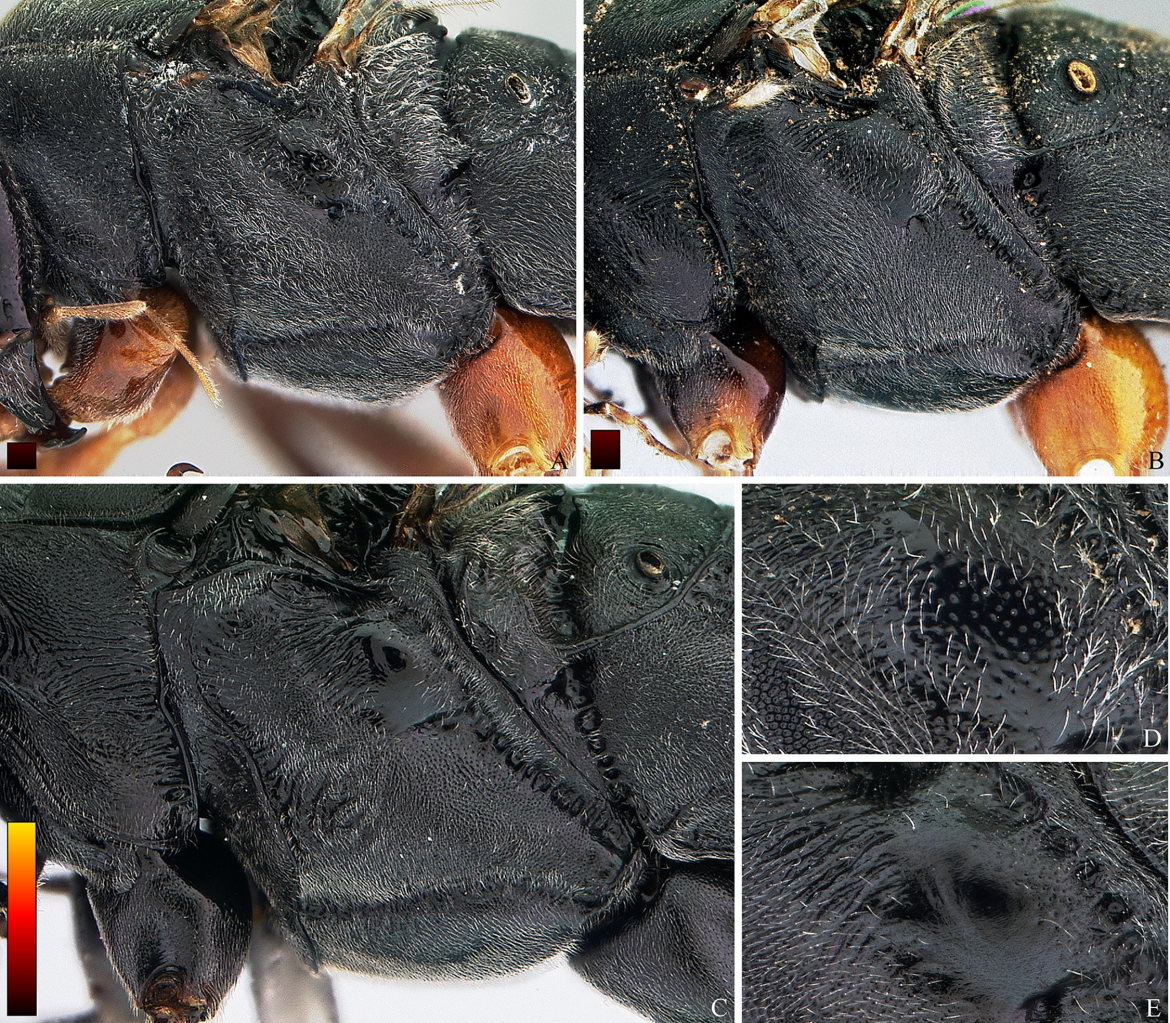
*Cryptoxenodon metamorphus* sp. nov. from the paratype series. **Female mesosoma.** (A-C) Mesosoma and propodeum (part), left side. (A) FAS4605, Alt. 75m, Colombia. (B) FAS4601, Alt. 111m, Brazil/AM. (C) FAS331, Alt. 561m, Brazil/ES. (D-E) Speculum on left side. (D) FAS4611, Alt. 36m, Brazil/PA. (E) UFES45548, Alt. 115m, Brazil/BA. Bars are proportional to the altitude.

**Fig 13:**
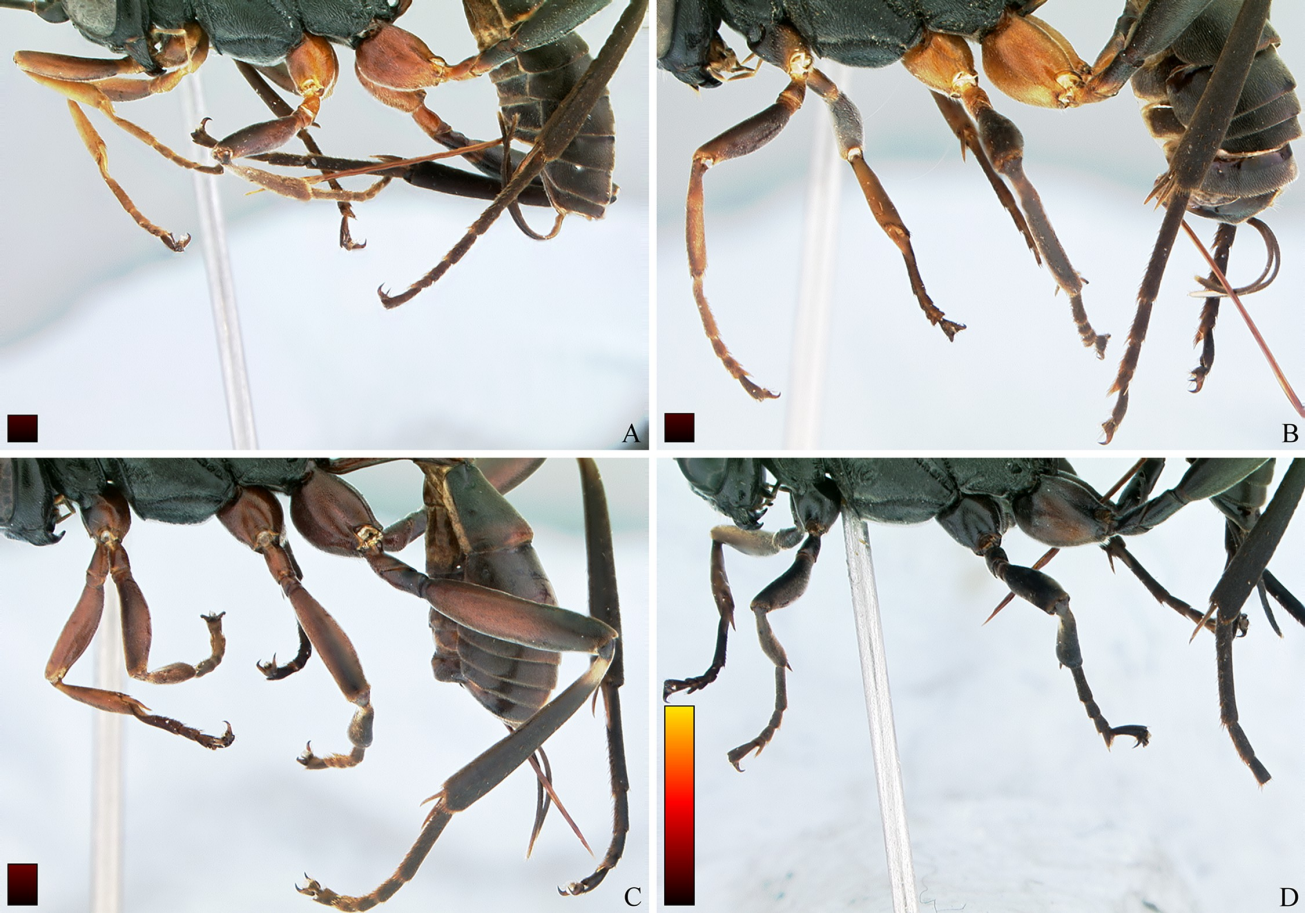
*Cryptoxenodon metamorphus* sp. nov. from the paratype series. **Female legs, left side.** (A) FAS4605, Alt. 75m, Colombia. (B) FAS4604, Alt. 80m, French Guiana. (C) UFES45548, Alt. 115m, Brazil/BA. (D) FAS331, Alt. 561m, Brazil/ES. Bars are proportional to the altitude.

**Fig 14:**
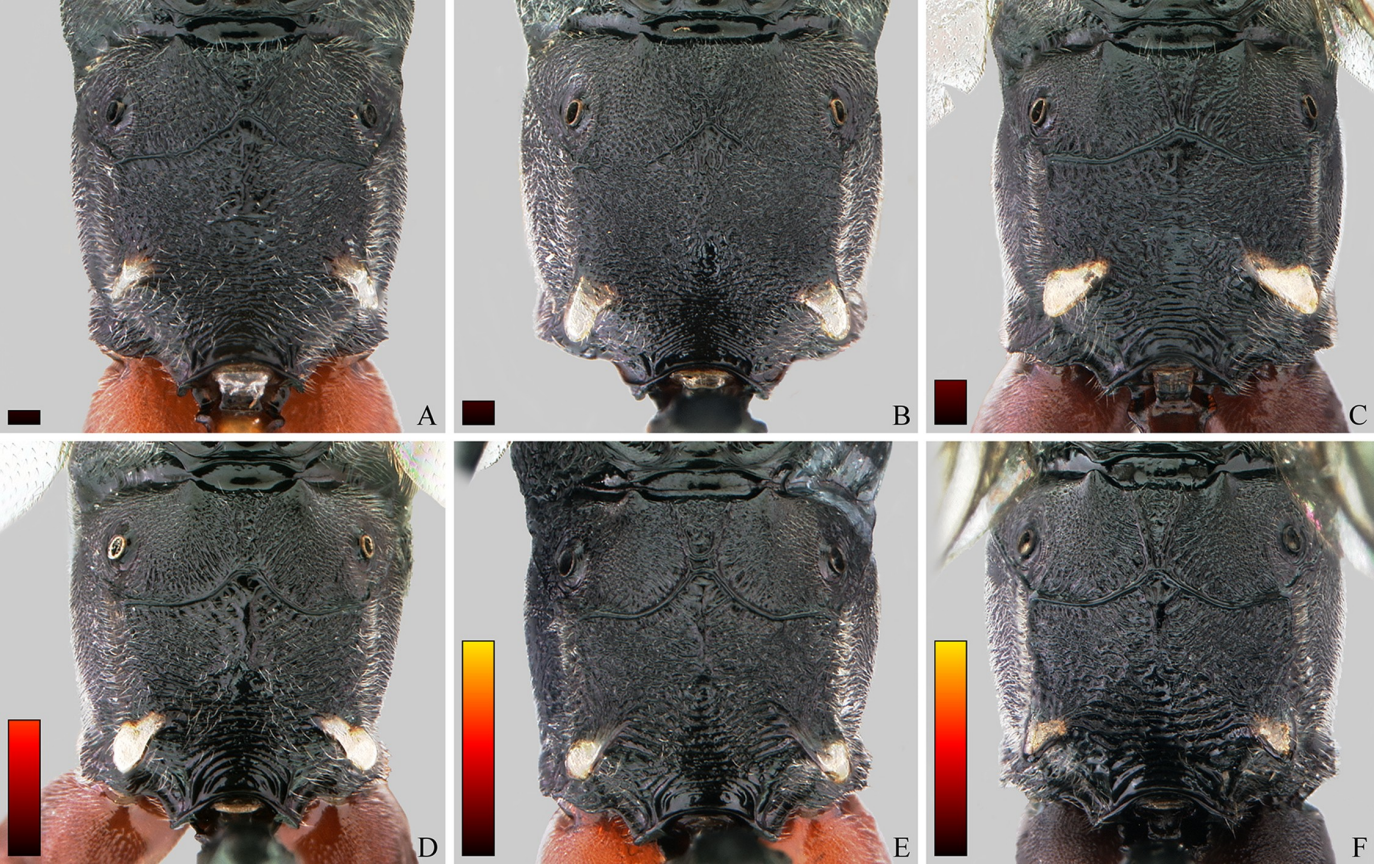
*Cryptoxenodon metamorphus* sp. nov. Female propodeum, dorsal view. (A) Paratype FAS4613, Alt. 36m, Brazil/PA. (B) Paratype, FAS4603, Alt. 61m, French Guiana. (C) Paratype UFES45548, Alt. 115m, Brazil/BA. (D) Paratype UFES49176, Alt. 354m, Brazil/BA. (E) **Holotype** FAS1127, Alt. 561m, Brazil/ES. (F) Paratype FAS331, Alt. 561m, Brazil/ES. Bars are proportional to the altitude.

**Fig 15:**
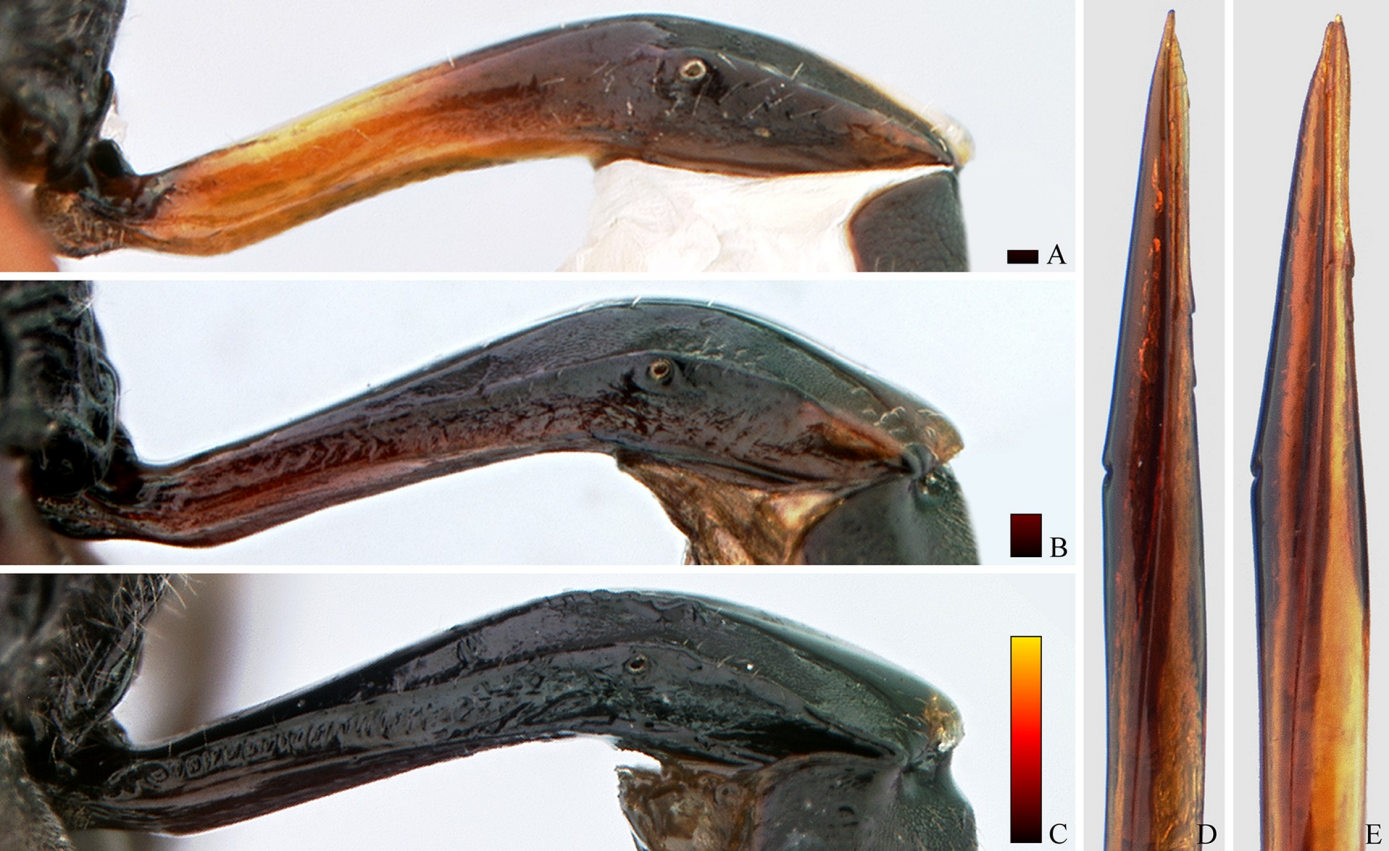
*Cryptoxenodon metamorphus* sp. nov. from the paratype series. Female petiole and ovipositor apex. (A-C), First tergite, left side. (A) FAS4613, Alt. 36m, Brazil/PA. (B) UFES45548, Alt. 115m, Brazil/BA. (C) FAS331, Alt. 561m, Brazil/ES. (D-E), apex of ovipositor, left side. (D) FAS333, Alt. 501m, Brazil/ES. (E) FAS4613, Alt. 36m, Brazil/PA. Bars are proportional to the altitude.

**Fig 16:**
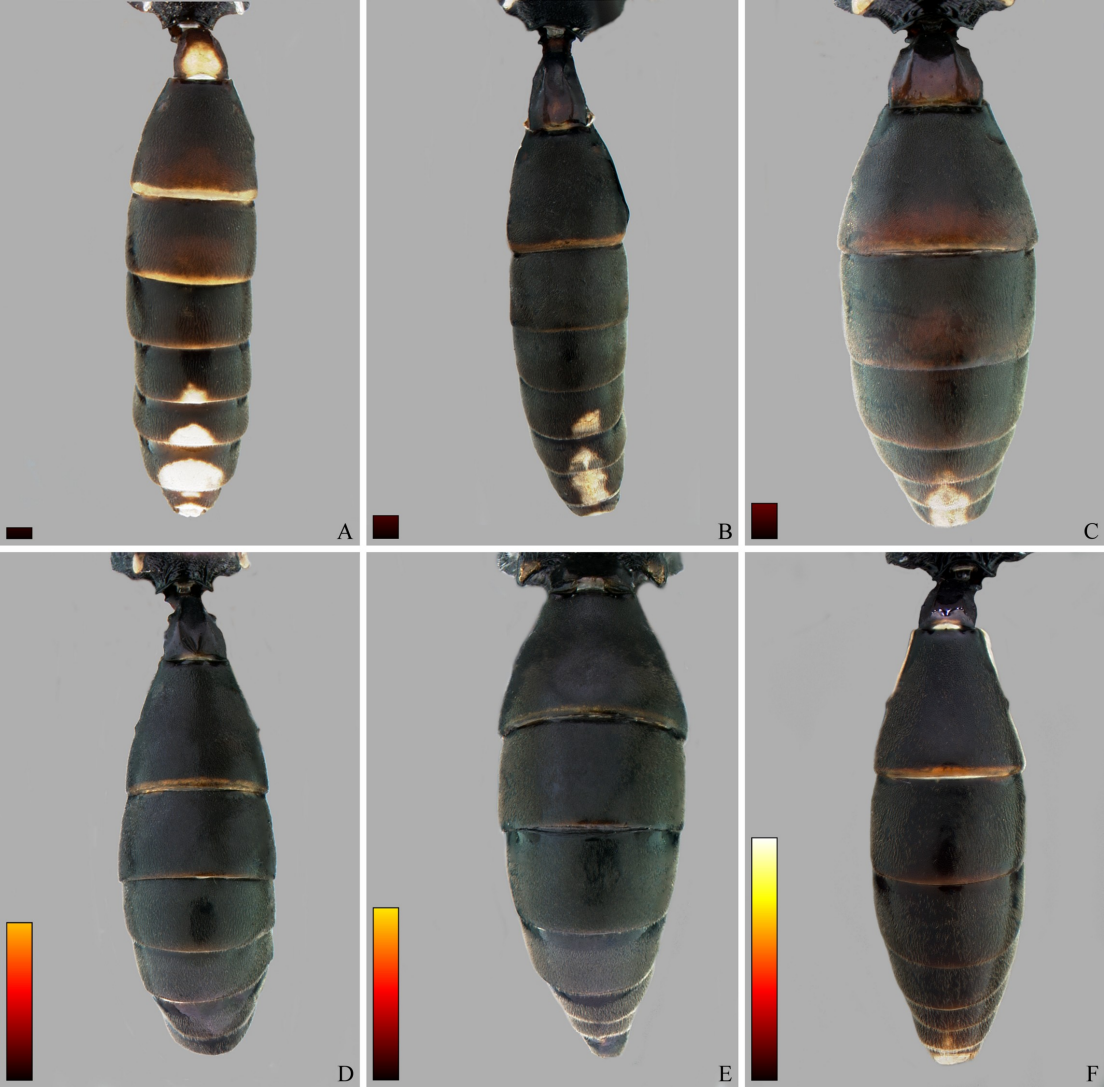
*Cryptoxenodon metamorphus* sp. nov. from the paratype series. Female metasoma, dorsal view. (A) FAS4606, Alt. 36m, Brazil/PA. (B) FAS4605, Alt. 75m, Colombia. (C) UFES45548, Alt. 115m, Brazil/BA. (D) FAS1165, Alt. 513m, Brazil/ES. (E) FAS331, Alt. 561m, Brazil/ES. (F) UFES41706, Alt. 790m, Brazil/ES. Bars are proportional to the altitude.

**Type species**. *Cryptoxenodon metamorphus* **sp**. **nov**., by present designation.

**Diagnosis.** Fore wing 5.85–9.25 mm long; body moderately stout (Fig 9). Head in frontal view subquadrate; clypeus quite wide, apical margin sharp, without apical tooth; mandibles very long, MLW 2.45–2.95 (Fig 10), basally distinctly concave; ventral tooth distinctly longer than dorsal one; gena ventrally about twice as wide as medially. Sternaulus complete, distinct and crenulate throughout; posterior margin of metanotum with distinct tooth-like projections; areolet moderately small, about as long as pterostigma width, closed (crossvein 3r-m spectral, sometimes nebulous); slightly wider than long; crossvein 2m-cu distinctly apical (2-M much longer than 3-M) (Fig 11B). Petiole rather long (Fig 9); anteriorly with distinct triangular projection on each side; spiracle near to posterior 0.35. Propodeum with distinct anterior transverse carina, without posterior transverse carina; sublateral apophyses conspicuously projected (Figs 11A and 14), about as long as second mid tarsomere. Ovipositor distinctly compressed, dorsal valve with nodus and pre-apical notch, ventral valve apex with few minute serrations (Fig 15D and 15E).

**Description.** Body surface matte. HEAD. Clypeus outline in lateral view distinctly convex on basal half, apical portion truncate; clypeal margin medially convex, even if slightly, without teeth, laterally without projections. Mandible ventral margin not projected as a flange or crest; ventral tooth longer than dorsal tooth. Flagellum subapical width enlarged, distinctly greater than the rest of flagellum; apical flagellomere apex regular, uniformly tapered. Supra-antennal area outline strongly concave throughout, medially with a low, suture-like median longitudinal line; supra-antennal horns or tubercles absent. Gena ventrally from about as wide as at eye midlength to somewhat swollen, distinctly wider than at eye midlength. Vertex dorsomedial outline without sulcus. Occipital carina dorsomedially present and conspicuous; ventrolaterally linear, not raised; ventral extension meeting hypostomal carina far dorsad of base of mandible. Hypostomal carina ventral outline low, linear, not forming a flange.

*THORAX*. Pronotum dorsal margin outline regular, not or only slightly swollen; central portion flat or slightly concave, without a transverse sulcus; pronotal collar posterior margin not bordered by a carina; epomia short or moderately long, very delicate to moderately strong, usually ending far from dorsal margin of pronotum. Notauli distinctly convergent; posterior end straight, not abruptly curved mesad; from moderately impressed and narrow to deeply impressed and wide; if deep and wide, distinctly carinate inside. Mesal lobe of mesoscutum without longitudinal groove. Scuto-scutellar groove crenulated. Epicnemial carina complete, reaching subalar ridge or nearly so; ventral outline, uniformly raised as a flange. Sternaulus complete and distinct throughout; anteriorly placed somewhat ventrally on mesothorax, facing downwards; crenulate. Postpectal carina vestigial, represented only by a short ridge on mesosternum; median portion arched anteriorward, somewhat v-shaped; ventral outline linear, without projections or flanges. Metapleural triangle distinct, not impressed. Posterior margin of metanotum with teeth-like projections. Transverse furrow at base of propodeum, smooth. Juxtacoxal carina present and distinct, even if incomplete. Pleural carina from weak and incomplete to distinct and complete. Fore tibia normal, not swollen or basally constricted; fourth tarsomeres distinctly bilobed, approximately of equal length. Hind fifth tarsomere without midventral bristles.

*PROPODEUM*. Anterior margin linear, with teeth-like projections or tubercles; anterior area outline regular, not raised, in lateral view at the same level as the rest of propodeum. Propodeal spiracle distinctly elliptic. Anterior transverse carina distinct, strongly bent anteriad at median portion. Posterior transverse carina absent; posterior sublateral portion of propodeum produced as a conspicuous tubercle. Median longitudinal carina absent. Lateral longitudinal carina absent.

*WINGS*. Forewing hyaline to moderately infuscate, sometimes with 1–2 dark spots, even if weak; short vein projection (ramellus) at junction of forewing veins 1m-cu and 1-Rs+M from completely absent to present, long; crossvein 1cu-a from basad to slightly apicad base of 1M+Rs; cell 2Cu (first subdiscal) approximately trapezoidal, apically distinctly longer than basally; vein 2Cua distinctly longer than crossvein 2cu-a, sometimes nearly the same length of 2cu-a; veins 2Cua and 2cu-a angled, even if slightly; crossvein 2m-cu distinctly convex, with one bulla, placed centrally; crossvein 3r-m entirely spectral or nebulous, almost indistinct; crossveins 2r-m and 3r-m parallel or nearly so; crossvein 2r-m about as long as crossvein 3r-m; vein 2-M distinctly longer than 3-M.

Hind wing vein M+Cu subapically distinctly convex; vein Cua distinctly longer than crossvein cu-a; veins Cua and 1-M forming approximately right angle; vein 2-Rs complete, reaching wing margin; vein 1-R1 undifferentiated; vein Cub complete, reaching wing margin or almost so; vein Cub apical half distinctly convex; vein 2-1A distinct, reaching more than half the way to wing margin.

*METASOMA*. First metasomal tergite (T1) moderately triangular, width at posterior apex over 2.10–3.00 × width at anterior apex; ventrolateral outline approximately rounded, giving the petiole ventrally somewhat cylindrical shape; anterolateral triangular tooth present; dorsal face with median depression, approximately at level of spiracle; spiracle placed distinctly posteriorly to midlength; spiracle in dorsal view prominent, protruding beyond tergite outline; dorsolateral carina complete, except weak to indistinct on smallest specimens; median dorsal carina distinct until the spiracle, or absent (smallest specimen); ventrolateral carina absent; postpetiole usualy not distinctly bent, at most forming weak angle with petiole; T1 subapically slightly wider than at posterior margin. Thyridium distinctly longer than wide or distinctly wider than long. T7–8 in lateral view of similar length, shorter than T5–6. Ovipositor in profile moderately slender, overall shape straight or nearly so, distinctly compressed. Dorsal valve without minute punctures throughout, its apical surface smooth; nodus moderately tall and distinct, giving triangular shape to apex; notch distinct; ovipositor tip moderately pointed. Ventral valve apex not projected dorsally as a lobe, smooth; tip with serrations, which are approximately vertical, uniformly arched.

**Male.** Moderately similar to females; most important morphological secondary sexual differences as follows. Body size almost always smaller than females, but the largest male specimens can be larger than the smallest females. Antenna typically with more flagellomeres (29–39) than in female (24–28), but the smallest specimens can have the same number of flagellomers as females (26–28); flagellum distinctly narrowing toward the apex, without a white band. Propodeal apophyses distinct but much smaller than in female; posterior transverse carina usually strong and irregular, medially distinctly arched forwards; area behind posterior transverse carina medially almost always with two strong and irregular longitudinal carinae; propodeal sculpture distinctly coarser than in female. First metasomal segment slender than in female, with T1LW 4.02–5.08 (vs. 1.70–2.20), and distinctly less widened apically, with T1WW 1.44–1.69 (vs. 2.10–3.00). There are also considerable differences in color patterns, as detailed in the description of the species.

**Comments.** *Cryptoxenodon* **gen**. **nov**. can be readily distinguished from all other known Cryptinae by the unusually stout, large mandibles (Fig 10), 2.45–2.95 as long as basal width, with the ventral tooth distinctly longer than the dorsal one. A diagnostic key is also provided below. There are several other cryptine genera with long mandibles, including all nine genera of Townes’ subtribe Osprynchotina (= Nematopodiina) and *Dotocrytpus* Brèthes. In these genera, however, the mandibles are almost always slender and distinctly tapered towards the apex, sometimes sickle shaped. Furthermore, in all those genera except for the distinctive *Dotocryptus* Brèthes, the dorsal tooth is longer than the ventral one.

It remains to be seen whether the large mandibles will hold as diagnostic for other eventual species in the genus. This could represent an isolated specialization, as with the markedly large and bent mandibles of *Gabunia flavitarsis* Kriechbaumer, the only species to show this trait out of eight known for the genus.

Specimens of *Cryptoxenodon* **gen**. **nov**. are otherwise similar to *Diapetimorpha* Viereck and will run to that genus in Townes’ key [7]. The new genus can be additionally separated from *Diapetimorpha* by having fore wing vein 2-M much longer than 3-M, i.e. the crossvein 2m-cu arises apicad to the middle of the areolet (vs. 2m-cu always basal or at midlength, 2-M smaller than or as long as 3-M); ventral tooth distinctly longer than dorsal tooth (vs. slightly shorter than dorsal tooth); clypeus nearly flat, confluent with supra-clypeal area (vs. rather evenly convex except somewhat flattened or impressed near apical margin); female flagellum subapical width enlarged, distinctly greater than the rest of flagellum (vs. regular, more or less uniform with rest of flagellum); supra-antennal area outline strongly concave throughout (vs. distinctly concave on ventral half); mesosoma more elongate, about 1.80 × as long as tall (vs. often shorter, around 1.50–1.60 × as long as tall). *Cryptoxenodon* **gen**. **nov**. is also similar to some species of *Debilos* Townes 1966, from which it can be differentiated by having the first tergite with an anterolateral tooth (vs. absent); ventral tooth distinctly longer than dorsal tooth (vs. about half of dorsal tooth length); and areolet closed, with crossvein 3r-m spectral or sometimes nebulous (vs. areolet open, 3r-m absent).

**Biology.** Unknown.

**Etymology.** From the Greek *xenos*, meaning strange, foreign, and the Greek suffix *-odon*, for tooth. The name is a loose reference to the unusual mandible, apparently unique within the Cryptinae.

**Distribution.** Recorded from French Guiana to southeastern Brazil (ES).

**Diagnostic key.** By using the mandible feature only at the end, the key below allows the user to further test any Neotropical Cryptini against the concept of ***Cryptoxenodon* gen**. **nov**.

1. Transverse furrow at base of propodeum very deep to moderately shallow… 2
  - Transverse furrow at base of propodeum very shallow, inconspicuous… Other 18 Cryptini genera
2. Sternaulus complete and distinct throughout… 3
  - Sternaulus complete but faint on posterior third OR incomplete, reaching 0.45–0.65 of the distance to middle coxa… Other 43 Cryptini genera
3. Vein 2Cua distinctly longer than crossvein 2cu-a… 4
  - Vein 2Cua nearly of the same length of 2cu-a, or 2cu-a slightly longer OR much shorter than crossvein 2cu-a, sometimes almost indistinct… Other 12 Cryptini genera
4. T1 anterolateral tooth absent… Other 6 Cryptini genera
  - T1 anterolateral tooth present, even if in the form of a triangular flange… 5
5. Propodeum sublaterally without distinct apophyses, at most with slight crest formed by raisig of posterior transverse carina… ***Mesostenus* Gravenhorst**
  - Propodeum sublaterally with distinct conical or spine-like apophysis, posterior transverse carina otherwise distinct or indistinct… 6
6. Areolet large, longer than greatest width of pterostigma. Propodeal spiracle elongate, SWL > 2.5. Mandible moderately short, MLW < 1.6… ***Xiphonychidion* Porter**
  - Areolet small, about as long as or shorter than greatest width of pterostigma. Propodeal spiracle short elliptic, SWL < 1.7. Mandible exceptionally long, MLW > 2.4… ***Cryptoxenodon* gen**. **nov**.

***Cryptoxenodon metamorphus* Supeleto, Santos & Aguiar, sp. nov.** (Figs 9–20)

**Fig 17:**
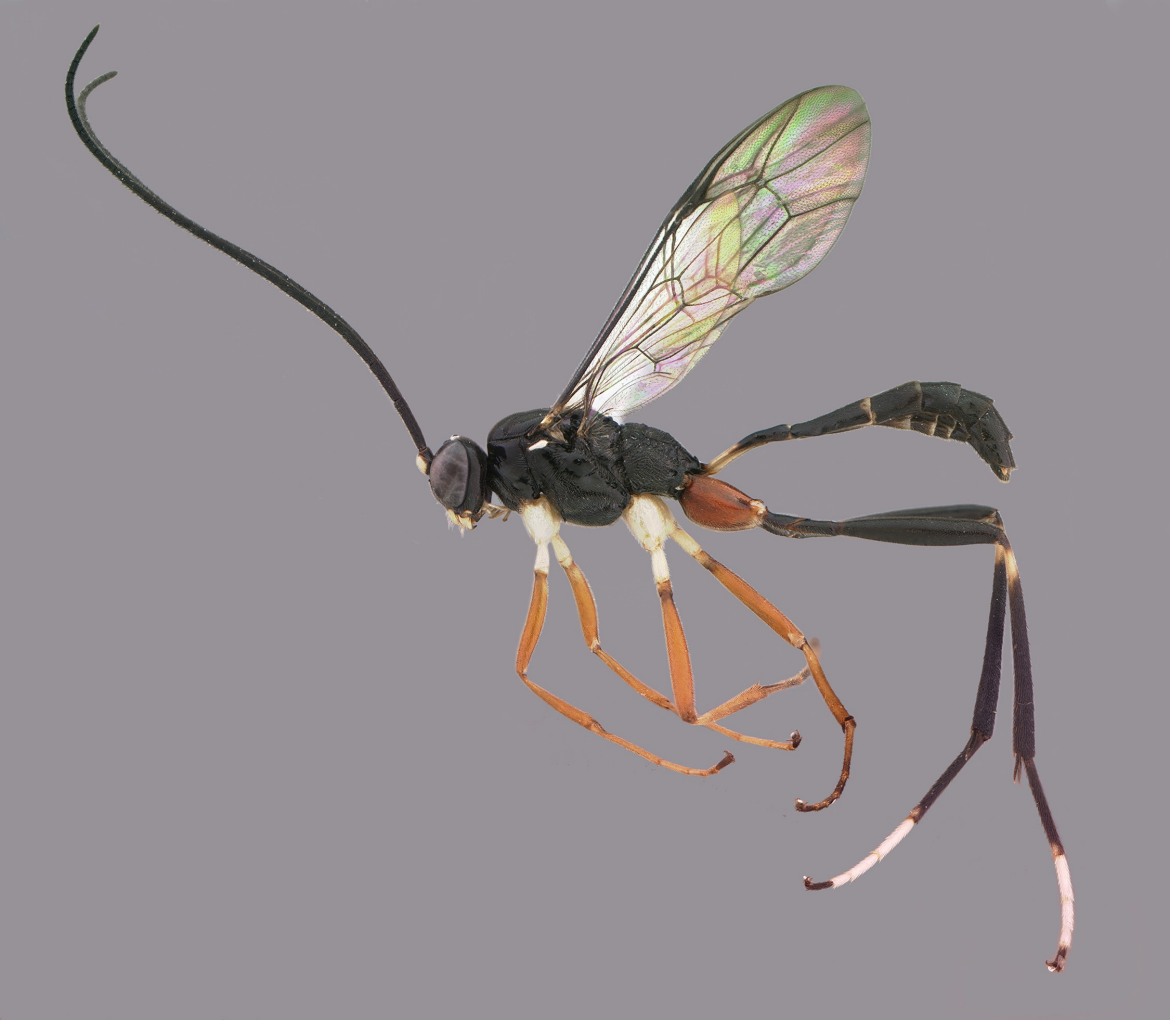
Paratype male of *Cryptoxenodon metamorphus* sp. nov. FAS2145, Alt. 501m, Brazil/ES. Habitus.

**Fig 18:**
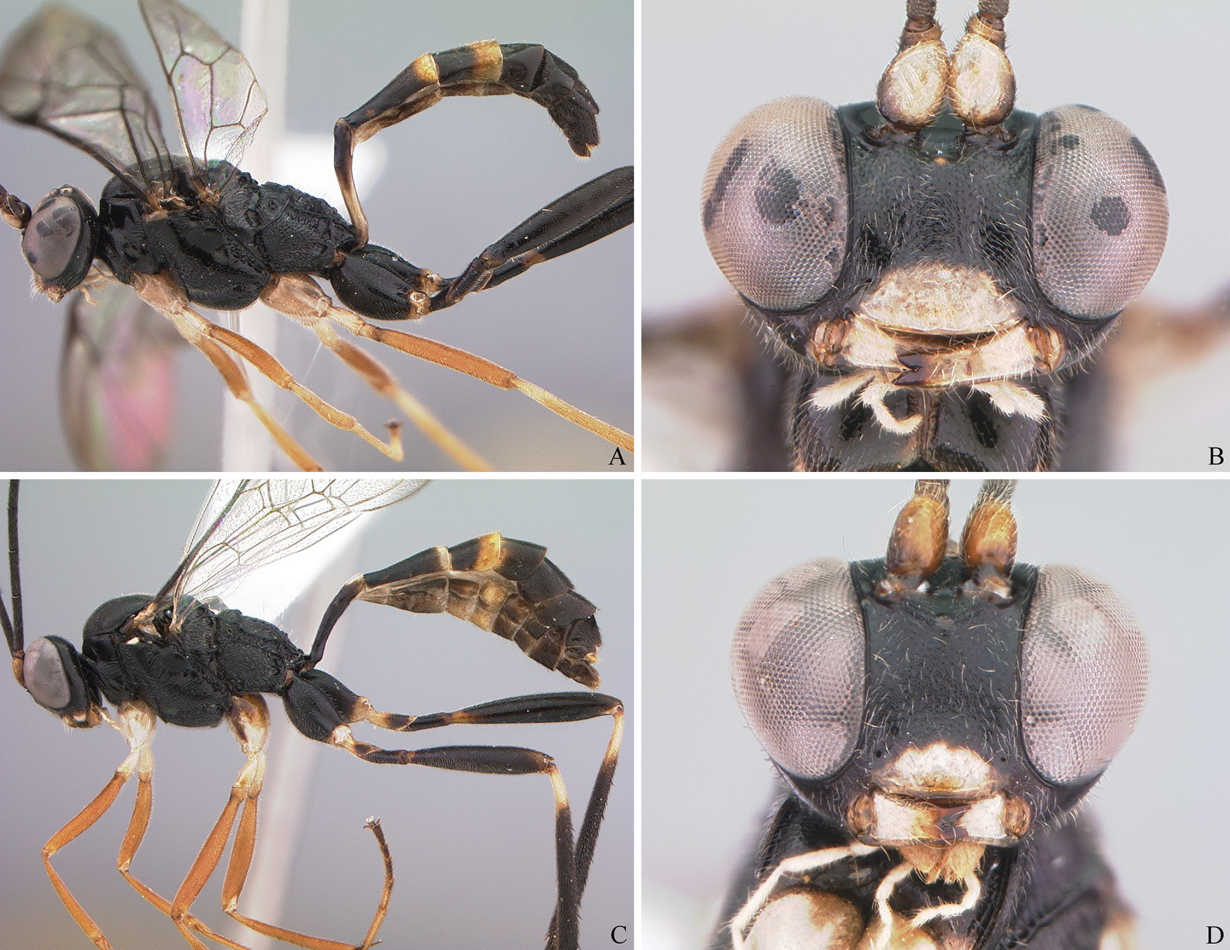
**Comparison between a male of *Cryptoxenodon metamorphus* sp. nov. (top) and a similar *Diapetimorpha* sp. (bottom).** The two specimens were collected from locations 155 Km apart. (A-B) Habitus and head in frontal view; paratype, UFES50549, Alt. 354m, Brazil/BA. (C-D) Habitus and head in frontal view; *Diapetimorpha* sp., UFES50346, Alt. 51m, Brazil/BA.

**Fig 19:**
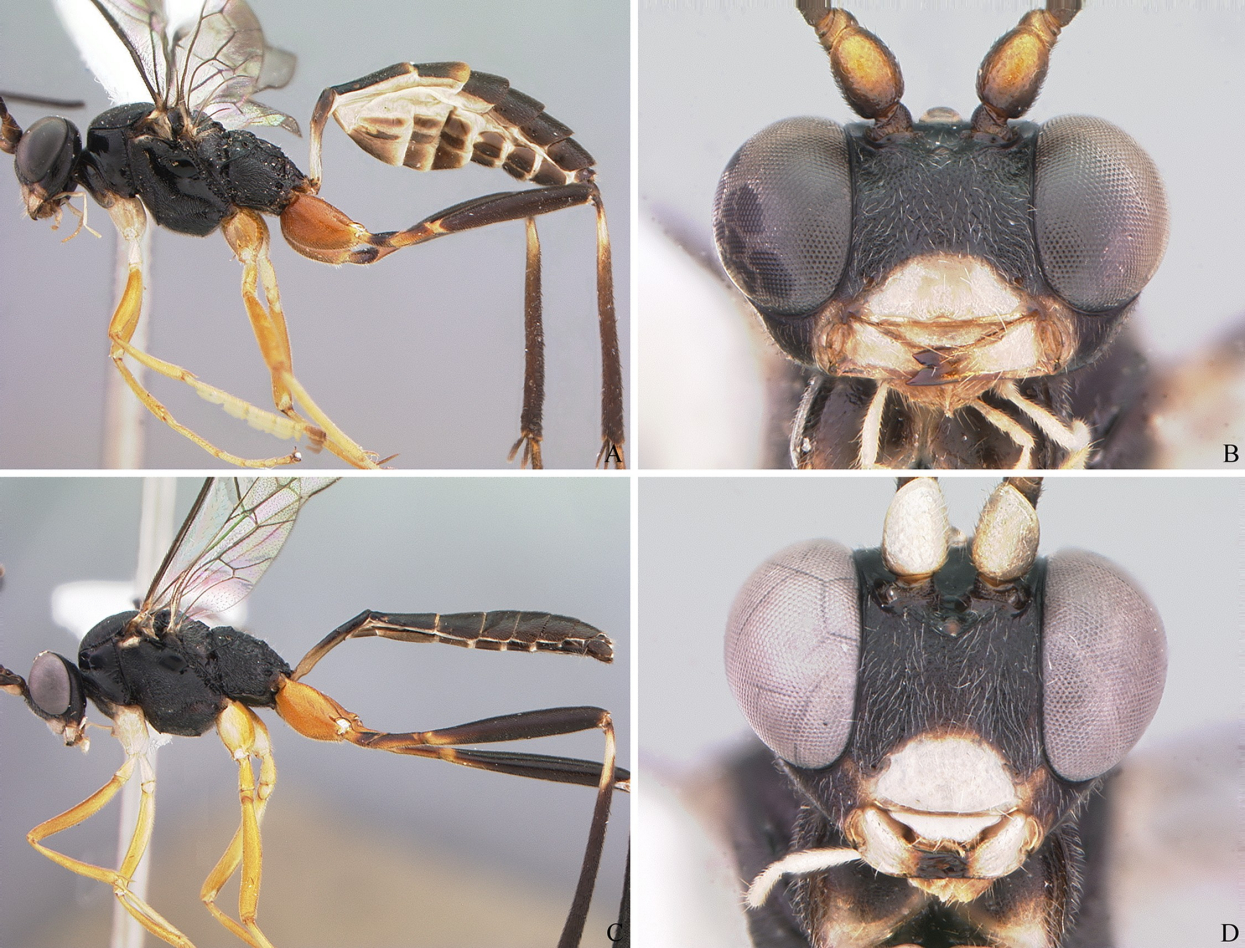
**Comparison between a male *Cryptoxenodon metamorphus* sp. nov. (top) and a similar *Diapetimorpha* sp. collected in the same reserve (bottom).** (A-B) Habitus and head in frontal view; paratype, FAS4618, Alt. 17m, Brazil/PA. (C-D), Habitus and head in frontal view; *Diapetimorpha* sp., FAS4619, Alt. 16m, Brazil/PA.

**Fig 20:**
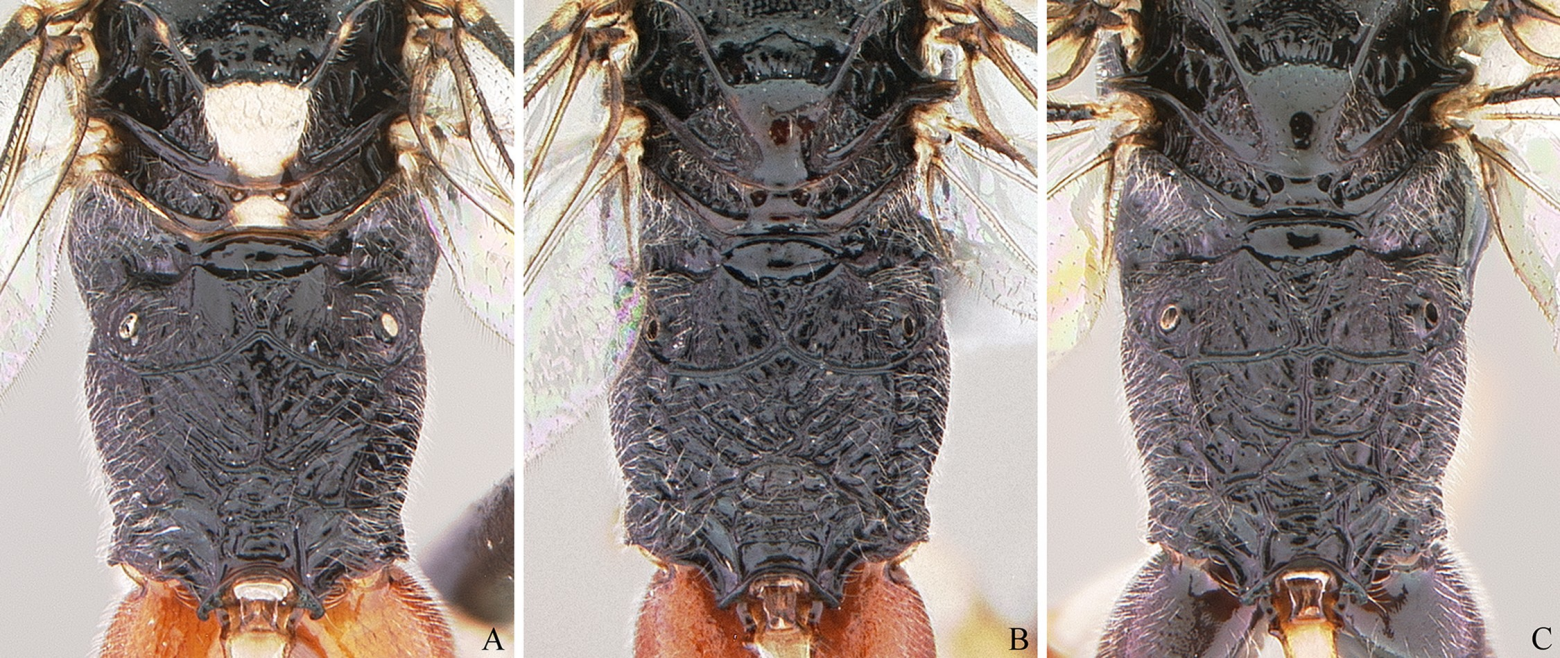
Males of *Cryptoxenodon metamorphus* sp. nov. from the paratype series. Propodeum, dorsal view. (A) FAS4620, Alt. 16m, Brazil/PA. (B) UFES49766, Alt. 150m, Brazil/BA. (C) UFES47242, Alt. 650m, Brazil/ES.

**Holotype** female. BRAZIL • ES, Cariacica, Duas Bocas, Alto Alegre, 20°17’26.4”S, 40°31’08.2”W; alt. 561 m; 20 Jan.–3 Feb. 2017; APAguiar & FASupeleto *leg*.; Mata Primária; Malaise; Pt1; UFES, FAS1127, Summer, sample No. 113. Complete, in good condition.

**Paratypes.** 51 females, 43 males. BRAZIL • 1 f; AM, Itacoatiara, Campus II, UFAM, 03°05’41.38”S, 58°27’33.43”W; alt. 37 m; 11 Nov. 2017; A. C. Barata e col. leg.; Malaise; Pt2; INPA, FAS4702, BRAZIL • 1 f; Manaus, EMBRAPA, Mata, 2°53’29.14”S, 59°58’45.8”W; alt. 97 m; 5 Jan. 2013; K. Schoeninger leg.; Border of *Paullinia cupana* organic culture; YPT; INPA, FAS4704 • 1 f; ibidem, except Reserva Florestal Adolfo Ducke, 2°57’48.30”S, 59°55’22.30”W; alt. 111 m; 1–3 Oct. 2005; APAguiar Exped.; Pt10; UFES, FAS4600 • 1 f; ibidem, except 10–12 Oct. 2005; Pt15; UFES, FAS4601 • 1 f; BA, Buerarema, Fazenda Boa Sorte, 14°56’49”S, 39°18’15”W; alt. 123 m; 29 Nov. 2002; JCardoso & JMaia leg.; Malaise; Pt.8; UFES, UFES49155 • 2 m; ibidem, except Fazenda Sempre Viva, 14°56’S, 39°18’W; alt. 150 m; 28 Nov. 2002; Pt6; UFES, UFES49766, UFES60330 • 1 m; ibidem, except Coaraci, Fazenda São José, 14°38’S, 39°32’W; alt. 214 m; 26 Nov. 2002; Pt3; UFES, UFES49108 • 1 m; ibidem, except Firmino Alves, Fazenda Bela Vista, 14°59’45”S, 39°55’50”W; alt. 350 m; 23 Nov. 2002; Pt2; UFES, UFES45663 • 1 m; ibidem, except Pt8; UFES, UFES45668 • 1 m; ibidem, except 9 Apr. 2003; Pt6; UFES, UFES49753 • 1 m; ibidem, except 11 Jun. 2003; Pt4; UFES, UFES50552 • 1 m; ibidem, except Fazenda Santo Antônio, 14°59’51”S, 39°55’55”W; alt. 354 m; 24 Nov. 2002; UFES, UFES50301 • 1 f; ibidem, except 9 Apr. 2003; Pt.3; UFES, UFES49176 • 1 m; ibidem, except 4 Apr. 2003; Pt7; UFES, UFES45936 • 2 m; ibidem, except 9 Apr. 2003; Pt2; UFES, UFES50549, UFES50551 • 1 m; ibidem, except Iguaí, Fazenda Montevideo, 14°42’34”S, 40°00’15”W; alt. 352 m; 10 Apr. 2003; Pt1; UFES, UFES49723 • 1 m; ibidem, except Ilhéus, Fazenda São José, 14°42’S, 39°11’W; alt. 111 m; 24 Nov. 2002; Pt7; UFES, UFES45864 • 2 m; ibidem, except 26 Nov. 2002; Pt2; UFES, UFES45874, UFES45872 • 1 f; ibidem, except 14°34’30”S, 39°12’00”W; alt. 89 m; 9 Dec. 2003; Pt.7; UFES, UFES45885 • 1 m; ibidem, except 14°42’S, 39°11’W; alt. 111 m; Pt4; UFES, UFES45218 • 1 m; ibidem, except Pt7; UFES, UFES45884 • 1 m; ibidem, except Pt2; UFES, UFES49722 • 1 m; ibidem, except 12 Apr. 2003; UFES, UFES45207 • 2 m; ibidem, except 14°47’37.28”S, 39° 2’46.65”W; alt. 60 m; 31 Dec. 2002; UFES, UFES59806, UFES59802, CEPLAC/CEPEC • 1 m; ibidem, except Itacaré, Fazenda Muchirão, 14°20’48”S, 39°18’38”W; alt. 94 m; 22 Nov. 2002; JCardoso & JMaia leg.; Malaise; Pt2; UFES, UFES45225 • 2 f; ibidem, except 12 Dec. 2003; Pt.8; UFES, UFES45852, UFES45855 • 2 m; ibidem, except Pt8; UFES, UFES45854, UFES45853 • 1 m; ibidem, except Pt4; UFES, UFES58952 • 1 m; ibidem, except 11 Apr. 2003; Pt7; UFES, UFES45237 • 1 f; ibidem, except 14°46’00”S, 39°29’00”W; alt. 52 m; 8 Feb. 2002; CEPLAC Exped.; L-11; UFES, FAS4614, Enlarged Malaise trap • 1 m; ibidem, except Uruçuca, Fazenda Guarani, 14°34’30”S, 39°19’47”W; alt. 210 m; 23 Nov. 2003; JCardoso & JMaia leg.; Pt2; UFES, UFES45539 • 1 m; ibidem, except Pt1; UFES, UFES49089 • 2 m; ibidem, except UFES, UFES49491, UFES58954 • 1 f; ibidem, except 14°42’41”S, 39°11’33”W; alt. 115 m; 12 Dec. 2003; Pt.2; UFES, UFES45548 • 1 m; ibidem, except 14°35’12”S, 39°17’28”W; alt. 97 m; 24 Nov. 2002; Pt8; UFES, UFES45507 • 3 m; ibidem, except 14°35’12.51”S, 39°17’28.98”W; Pt3; UFES, UFES49119, UFES49117, UFES49116 • 1 m; ibidem, except 14°35’12.5”S, 39°17’29.0”W; Pt1; UFES, UFES49170 • 2 m; ibidem, except 14°35’15.5”S, 39°17’28.0”W; Pt5; UFES, UFES50368, UFES50369 • 2 f; ES, Alfredo Chaves, Picadão (mata), 20°27’53”S, 40°42’35”W; alt. 714 m; 8–15 Oct. 2007; COAzevedo Exped.; 5; UFES, UFES49039, UFES49049 • 1 f; ibidem, except 8; UFES, UFES50636 • 1 f; Cariacica, Duas Bocas, Alto Alegre, 20°17’26.4”S, 40°31’08.2”W; alt. 561 m; 5–20 Oct. 2016; APAguiar & FASupeleto leg.; Mata Primária; ibidem; Pt1; UFES, FAS331, Spring, sample No. 39 • 1 f; ibidem, except 20°16’54.4”S, 40°31’20.7”W; alt. 501 m; Pt6; UFES, FAS333, Spring, sample No. 44 • 1 f; ibidem, except 20°17’19.9”S, 40°31’19.3”W; alt. 557 m; 21–22 Jan. 2017; 40YPTs; Pt3; UFES, FAS332, Summer, sample No. 83 • 1 f; ibidem, except 20 Jan.–3 Feb. 2017; Malaise; Pt2; UFES, FAS1131, Summer, sample No. 115 • 1 f; ibidem, except 10–11 Apr. 2017; 40YPTs; Pt3; UFES, FAS1124, Autumn, sample No. 127 • 1 f; ibidem, except 20°16’54.4”S, 40°31’20.7”W; alt. 501 m; Pt6; UFES, FAS1126, Autumn, sample No. 130 • 1 f; ibidem, except 20°17’02.6”S, 40°31’20.1”W; alt. 513 m; Pt5; UFES, FAS1165, Autumn, sample No. 129 • 1 f; ibidem, except 10–26 Apr. 2017; Malaise; UFES, FAS334, Autumn, sample No. 141 • 3 f; ibidem, except Sede, 20°15’33.0”S, 40°29’21.8”W; alt. 185 m; 7–21 Oct. 2016; Mata Secundária; UFES, FAS335, FAS1125, FAS1130, Spring, sample No. 55 • 2 m; ibidem, except Reserva Biológica de Duas Bocas, Alto Alegre, 20°16’54.4”S, 40°31’20.7”W; alt. 501 m; 10–26 Apr. 2017; Mata Primária; Pt6; UFES, FAS2145, FAS2144 • 1 m; Domingos Martins, Mata Pico do Eldorado, 20°22’17”S, 40°39’29”W; alt. 701 m; 3–10 Dec. 2004; MTavares & eq. leg.; ibidem; B2; UFES, UFES43754 • 1 f; ibidem, except Rio Novo do Sul, Sítio Bortoloti, Comunidade São Vicente, 12–17 Oct. 2007; FRampinelli & MAraújo leg.; Pt03; UFES, UFES41966 • 1 f; ibidem, except Santa Maria de Jetibá, Fazenda Paulo Seick, 20°02’31.1”S, 40°41’51.3”W; alt. 790 m; 29 Nov.–6 Dec. 2002; MTavares & CAzevedo leg.; B2; UFES, UFES41706 • 1 f; ibidem, except 6–13 Dec. 2002; UFES, UFES41704 • 1 f; ibidem, except T2; UFES, UFES41705 • 1 f; Santa Teresa, Augusto Ruschi, Morro, 19°53’41.0”S, 40°32’45.0”W; alt. 789 m; 16 Apr.–1 May 2017; APAguiar & FASupeleto leg.; Mata Secundária; ibidem; Pt3; UFES, FAS1128, Autumn, sample No. 170 • 1 f; ibidem, except Trilha da Cachoeira, 19°55’16.4”S, 40°33’13.5”W; alt. 775 m; 8–24 Oct. 2016; Mata Primária; UFES, FAS1129, Spring, sample No. 67 • 1 m; ibidem, except Estação Biológica de Santa Lúcia, 19°57’54.95”S, 40°32’25.73”W; alt. 650 m; 9–13 May 2006; MTavares, CAzevedo & eq. leg.; UFES, UFES47242 • 1 f; PA, Belo Monte, Rio Xingu, Rodovia Transamazônica, 03°05’52”S, 51°41’31”W; alt. 41 m; 7 Apr. 2008; J. A. Rafael & F. F. Xavier Filho leg.; Light trap; INPA, FAS4701 • 1 f; Melgaço, Estação Cient. Ferreira Pena-Trilha, Trilha Igarapé Ararua, 1°45’59.8”S, 51°31’24.1”W; alt. 16 m; 24–27 Nov. 2003; APAguiar & JDias leg.; ibidem; YPT; P05180; MPEG, FAS4608 • 1 f; ibidem, except Trilha Igarapé Tijucaquara, 1°44’12.8”S, 51°29’56.6”W; alt. 17 m; 24–26 Nov. 2003; Malaise; P05189; MPEG, FAS4607, Malaise M11 • 1 m; ibidem, except 24–27 Nov. 2003; YPT; P05192; UFES, FAS4618 • 1 f; ibidem, except Trilha principal, 1°44’14.0”S, 51°27’23.0”W; alt. 36 m; 16–19 Nov. 2003; P05052; MPEG, FAS4606 • 1 f; ibidem, except P05054; MPEG, FAS4609 • 1 f; ibidem, except P05051; MPEG, FAS4610 • 2 f; ibidem, except 13–16 Nov. 2003; P05009; MPEG, FAS4612, FAS4611 • 1 f; ibidem, except 16–19 Nov. 2003; P05048; MPEG, FAS4613 • 1 m; Vitória do Xingu, Rio Xingu, Caracol, 2°53’15.54”S, 52° 0’44.10”W; alt. 16 m; 20–23 Nov. 2007; ibidem; UFES, FAS4620, M1 • 2 f; RR, Amajari, Serra do Tepequém, SESC, 03°44’45”N, 61°43’40W; alt. 416 m; 14–29 Dec. 2015; R. Boldrini & J.A. Rafael leg.; Malaise; INPA • 1 f; ibidem, except 1–5 Feb. 2016; INPA • 1 f; ibidem, except 1–15 Feb. 2016; INPA.

COLOMBIA • 1 f; Departamento de Amazonas, Leticia, Corregimiento La Pedrera, rio Ayo, 1°55’00”S, 69°31’00”W; alt. 75 m; 16–20 Jun. 2002; FQuevedo leg.; UFES, FAS4605, M3265

FRENCH GUIANA • 1 f; Eaux Claires, 3.5 mi N Saül, in forest, 03°39’N, 53°13’W; alt. 200 m; May-Nov. 1995; A. Berkov & D. Grimaldi leg.; AMNH, AMNH_IZC_00177986 • 1 f; 4°32’39.8”N, 52°09’22.3”W; alt. 230 m; Dec. 2007; J.Cerda leg.; Rainforest; PK35; CNCI, FAS4703, DNA voucher • 1 f; Nouragues, Sout-Pararé Camp, 4°02’00”N, 52°41’00”W; alt. 80 m; Nov. 2009; S.E.A.G. leg.; UFES, FAS4604 • 1 f; Roura, Montagne des chevaux, 4°43’22.22”N, 2°24’45.24”W; alt. 61 m; 22 Dec. 2008; UFES, FAS4602, YVES • 1 f; ibidem, except 1–31 Jul. 2009; UFES, FAS4603, YVES.

VENEZUELA • 1 f; Amazonas state; M[oun]t [Cerro] Duida; 3°30’48”N, 65°37’34”W; alt. 1135 m; 4 Nov. 1928; G. H. H. Tate; AMNH, Tate No. 156 / Ac. 29500.

**Description.** Female holotype (Figs 9, 10D, 11A and 14E): Fore wing 9.05 mm long. Body stout. HEAD (Figs 9, 10 and 11A). In frontal view wide, somewhat squared; in lateral view distinctly higher (1.55 ×) than long. Mandible quite large, about as long (1.05 ×) as inter-ocular distance at level of antennal foramen; basal half externally deeply and widely concave, progressively shallower until concavity disappears near middle; elongate, MLW 2.65, narrowing considerably towards apex, MWW 0.55; ventral tooth 1.35 × longer than dorsal one. Malar space relatively short because of large mandible, MSM 0.55. Clypeus large, very wide, CHW 0.35, nearly flat, separated from supra-clypeal area by distinct transversal suture; dorsal third punctulated, centrally transversely rugulose, apical third nearly smooth; apical margin sharp, almost straight. Supraclypeal area densely punctulated, medially prominent. Antenna with 26 flagellomeres; flagellum basally narrow, f1–3 much longer (6.20, 5.30, 4.50 ×) than wide, compressed, height 1.15 × width, f1–6 relative size 3.1:2.7:2.4:1.6:1.1:1.0; remaining of flagellum thick, f4–24 short and wide, length/width from f4 = 2.60 to f20 = 0.40; apical flagellomere bullet-shaped, apex with quite short, stiff, blunt setae. Occipital carina distinct, sharp throughout, meeting hypostomal carina far from mandible base. Gena ventrally about twice as wide as at level of eye midlength.

THORAX (Figs 9, 11A, 12 and 13). Pronotum mostly coarsely sculptured, from dorso-laterally punctulate-reticulate to mid-laterally strigose, which is stronger towards posterior margin, strigose extending to ventral lobe posteriorly only; anterior margin raised, punctulate; epomia indistinct. Mesoscutum moderately convex, ovoid, 1.10 × as long as wide; matte, densely punctulate to punctulate-reticulate; notauli very deeply impressed, delicately crenulate, long, meeting posteriorly on the 0.75 of mesoscutum length; scuto-scutellar groove very deep, with 8 stout longitudinal carinae. Scutellum sparsely punctulate, area in between punctures smooth and shiny. Postscutellum with two quite deep, round fovea, one on each side. Subalar ridge strongly raised, elongate. Epicnemial carina reaching subalar ridge. Mesopleuron centrally densely, finely punctulate; both in front and dorsally to speculum with coarse transversal rugosity; anteriorly, on ventral half, with an area of coarse rugosities which are continuous with strong rugosity/crenulations along epicnemial carina; speculum round, large, swollen, posteriorly punctate, centrally smooth and shiny, anteriorly receiving strigation from mesopleuron; scrobe quite deep; mesopleural groove strongly crenulate. Postpectal carina mostly indistinct. Metapleuron matte, densely, finely punctulate-areolate, posterior end with deep, irregular fovea. Hind margin of metanotum with low, tooth-like projections, shaped as right triangle; corresponding projections of propodeum strong, conical, acute. Transverse furrow at base of propodeum narrow, deep, smooth and shiny. Pleural carina strong, nearly straight; both pleural fovea and metapleural fovea deep; interfoveolar suture strongly crenulate. All fourth tarsomeres distinctly bilobed, lobes long, of subequal length, apically with distinct cluster of 4–6 stout, spiked setae; other tarsomeres not bilobed.

PROPODEUM (Figs 11A, 12A–12C and 14). Somewhat short, as long as wide, glabrous, coarsely and richly sculptured. Anterior transverse carina (ATC) with two diverging carinae arising centrally and reaching propodeal furrow; area anterior to ATC from coarsely rugulose to microareolate near spiracle; area behind ATC from transversely to somewhat concentrically, coarsely, areolate-rugose; median longitudinal carina weakly distinct from ATC to level of apophyses, partially confluent with surrounding rugosities; lateral longitudinal carinae partially distinct, confluent with surrounding rugosities; propodeal area sloped at about 90 degrees at the level of this carina. Propodeal spiracle elliptic, SWL 1.30. Propodeal tubercles large, subtriangular, about same length as second mid tarsomere.

WINGS (Figs 9 and 11B–11C). Fore wing 1-Rs+M with bulla placed distinctly before its midlength; ramellus quite short but distinct; crossvein 1cu-a distinctly basad (right wing) or opposite origin of 1M+Rs; vein 2Cua 1.25 × as long as 2cu-a; bulla at crossvein 2m-cu short, about 1/5 of the crossvein length; cell 1+2Rs (areolet) moderately small, APH 0.95, transverse pentagonal, distinctly wider than long, AWH 0.75; crossvein 2m-cu reaching areolet apically, vein 2-M 2.25 × as long as vein 3-M. Hind wing vein Cua 2.70 × length of crossvein cu-a; crossvein 1r-m with bulla at ventral 1/4; veins 1-Rs and 2-Rs weakly angled, cell R1 elongate; 9 hammuli; vein Cub at straight angle with Cua; vein 2-1A reaching 0.70 of distance to wing margin.

METASOMA (Figs 9, 11A, 13, 15 and 16). T2–8 densely pilose with short, velvety, golden hairs; T1 long, about 0.40 × as long as T2–8, petiole slender, T1LW 1.95, apex much wider than base, T1WW 3.00; T1 dorsally and laterally distinctly and widely concave at level of spiracles; T1 spiracle placed on posterior 0.40 of tergite; median dorsal carina strong from base to level of spiracle; dorsolateral and median dorsal carinae strong, distinct throughout. T2 short, T2LW 0.90, campanulate, apex much wider than base, T2WW 1.75; thyridium shallow, longitudinal. Ovipositor moderately long, OST 0.70; ventral valve apex with 6, minute serrations.

*COLOR*. Blackish with mid and hind coxae dark orange. Antennae with central white stripe, taking mostly dorsal and mesal sides of flagellomeres 5–11. Fore leg, except coxa, dark brown. Wings light amber infuscate. Propodeal apophyses whitish. Ovipositor dark orange.

**Variation** (female) (Figs 10, 12, 13, 14, 15A–15C and 16). The morphological variation is extensive and related to both altitudinal and latitudinal clines. In general, low altitude specimens (below 500 m) tend to show lighter tones, with orange on legs and metasoma, and T5–7 mid-apically sometimes with whitish spots, while high altitudes lead to nearly fully dark specimens, but the full range of variation is complex (Table 4; see also item Discussion).

Other variations are as follows. Antenna with 24–28 flagellomeres; fore wing 5.85–9.25 mm; HWHM 6–9. On the pronotum, the most typical pattern is laterally punctulate in its central, convex area, then anteriorly transversely strigate, and ventrally mostly polished smooth; this sculpturing structure holds for most specimens, but will vary as (1) very fine, matte (e.g., specimen from Colombia), or become (2) weak (e.g., specimen FAS4613) to inconspicuous, or (3) very strong (e.g., FAS331, with convex area punctulate-rugulose, and ventral area longitudinally carinate-rugose) (Fig 12A–12C). When the apices of the ovipositor dorsal and ventral valves are aligned it is possible to notice, in some specimens, that the nodus (dorsal valve) and the pre-apical swelling of the ventral valve, combine to make this part distinctly diamond-shaped (Fig 15D–15E). The ventral valve apex has 5–8, minute serrations. Areolet shape sometimes subpentagonal or subquadrate.

In nearly all specimens the anterior mid-longitudinal carinae of propodeum is V-shaped, but in specimens from Santa Maria de Jetibá (ES) it is distinctly Y-shaped. The posterior transverse carina is sometimes present only medially as a strong wrinkle, which may also be connected with the short mid-longitudinal carina (Fig 14A); more rarely, the posterior transverse carina occurs as a weak wrinkle extending all the way between the apophyses.

Morphological ratios varying as follows: AAW 0.85–1.20, APH 0.65–0.95, AWH 0.75–1.00, CHW 0.30–0.40, HWIA 0.60–0.80, HWIC 2.65–4.25, MELW 0.95–1.10, MLW 2.45–2.95, MSM 0.45–0.65, MWW 0.55–0.60, NLML 0.45–0.75, OST 0.65–0.75, RCUA 0.90–1.35, SPR 0.65–0.65, SWL 1.25–1.65, T2LW 0.75–1.00, T2WW 1.75–2.05, T1LW 1.70–2.20, T1WW 2.10–3.00.

**Male** (Figs 17, 18A–18B, 19A–19B, 20). Generally smaller, fore wing 4.90–7.10 mm (vs. 5.85–9.25); mandible more delicate, and usually not as long as that of female, but distinctly elongate, MLW 2.20–2.65 (vs. 2.45–2.95); flagellum narrowing toward the apex, without a white band; clypeus distinctly convex; pronotum mostly impunctate and shiny, almost always striate along posterior margin; posterior transverse carina of propodeum usually complete, strong and irregular, medially distinctly arched forwards; propodeal apophyses distinct, but much smaller than in female; area behind posterior transverse carina medially with two strong and irregular longitudinal carinae; propodeum sculpturing generally more coarser than in females. Color differences: fore wing hyaline, apical margin light infuscate, without darkened spots; mandibles, clypeus, apex of gena at base of mandible, scape ventrally, subalar ridge, fore and mid coxae, and fore and mid trochanters, whitish or pale yellow. Fore and mid femora light orange; fore and mid tibiae yellowish; fore and mid tarsi yellowish with the apical segment darkened. Hind tibia on basal 0.3 whitish. Hind t1 apically, t2–4, t5 basally, whitish. T2 and T3 blackish, posterior margin with wide yellow stripe.

**Variation** (male) (Figs 18A–18B, 19A–19B, 20). Antenna with 26–32 flagellomeres; posterior transverse carina of propodeum medially sometimes briefly interrupted; longitudinal carinae of propodeum arising from ATC towards propodeal sulcus varying in shape as in female; in about 65% of the specimens propodeum with a short mid-longitudinal carina between anterior and posterior transverse carinae (Fig 20C); scutellum sometimes with a posterior or central whistish spot, or more rarely entirely whitish (Fig 20A); scutelar carina, postscutellum and propodeal apophyses sometimes whitish; three specimens from PA and three from BA with T4 extensively yellow; posterior yellow stripes of T2 and T3 sometimes narrow and almost indistinct; T4 and T5 rarely with a yellow stripe on anterior margin, or with yellow marks posteriorly; colour pattern of hind coxa varying from light orange to blackish (Figs 17, 18A, 19A).

**Comments.** Males of *C*. *metamorphus* **sp**. **nov**. can be easily confused with males of a number of other Cryptinae taxa that have generally similar color pattern, in particular species of *Diapetimorpha*, which also show an anterolateral tooth on the T1; males of that genus also often show different color parttern from the respective females and remain unassociated in most cryptine samples. Males of *C*. *metamorphus* **sp**. **nov**. can also be mistaken for males of *Basileucus* Townes, for which a diagnostic feature is having the T1 anteriorly whitish. Nonetheless, males of *C*. *metamorphus* **sp**. **nov**. can be differentiated from these other taxa by having the ventral tooth of the mandible distinctly longer than the dorsal one (vs. slightly to distinctly shorter); mandible long and generally similar in shape and structure to that of the female, MLW > 2.20 (vs. short to moderately long, MLW < 2.00); and the areolet usually distinctly wider than long (vs. about as long as wide) (see Figs 18 and 19).

**Etymology.** From the Greek *meta*, meaning over, beyond, and the Greek *morphus*, meaning form, shape; a reference to the extreme morphological variation of the species.

**Fig 21:**
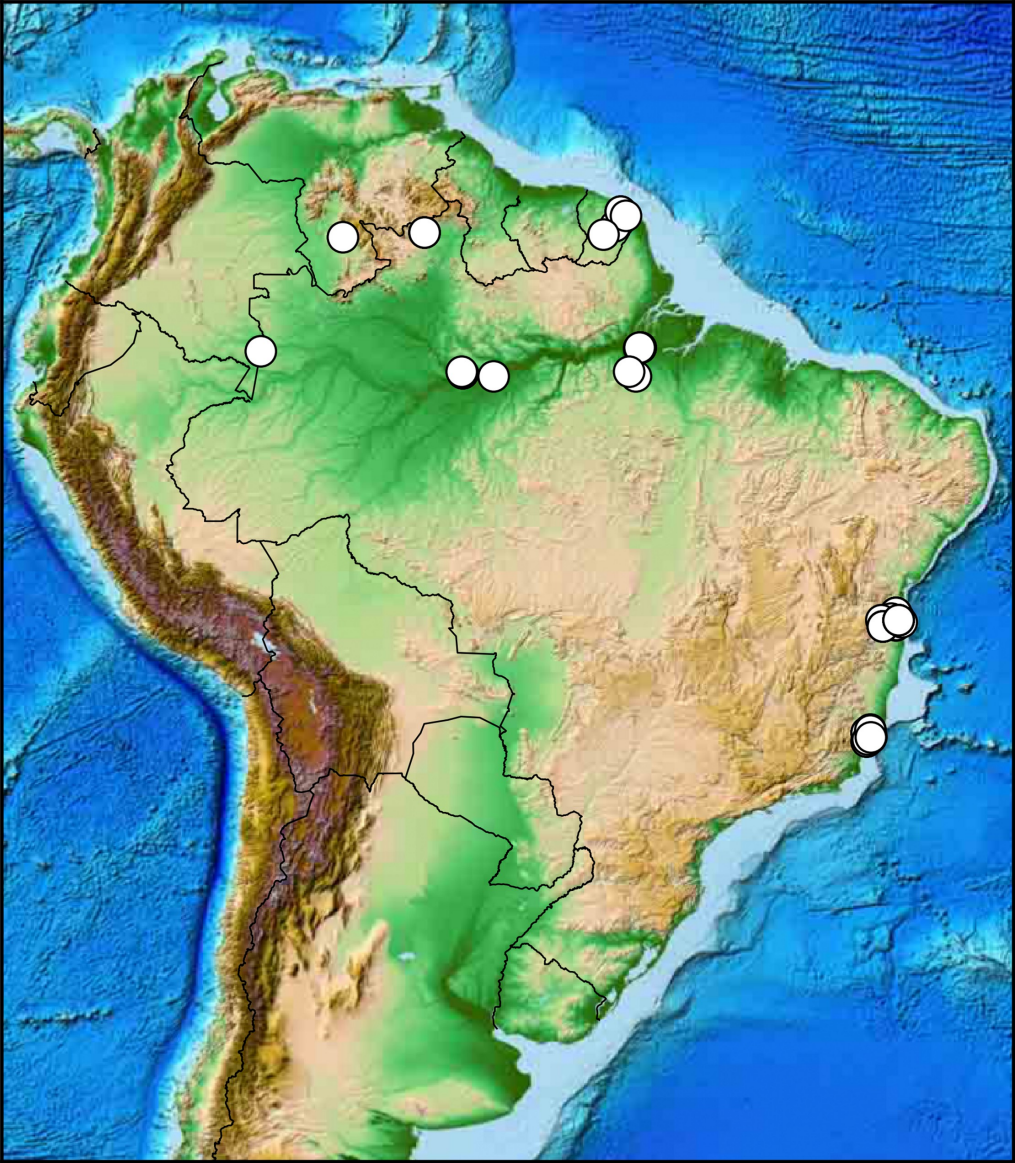
Distribution records for *Cryptoxenodon metamorphus* sp. nov. Terrain and vegetation projection. Built with Basemap (https://matplotlib.org/basemap/).

**Fig 22:**
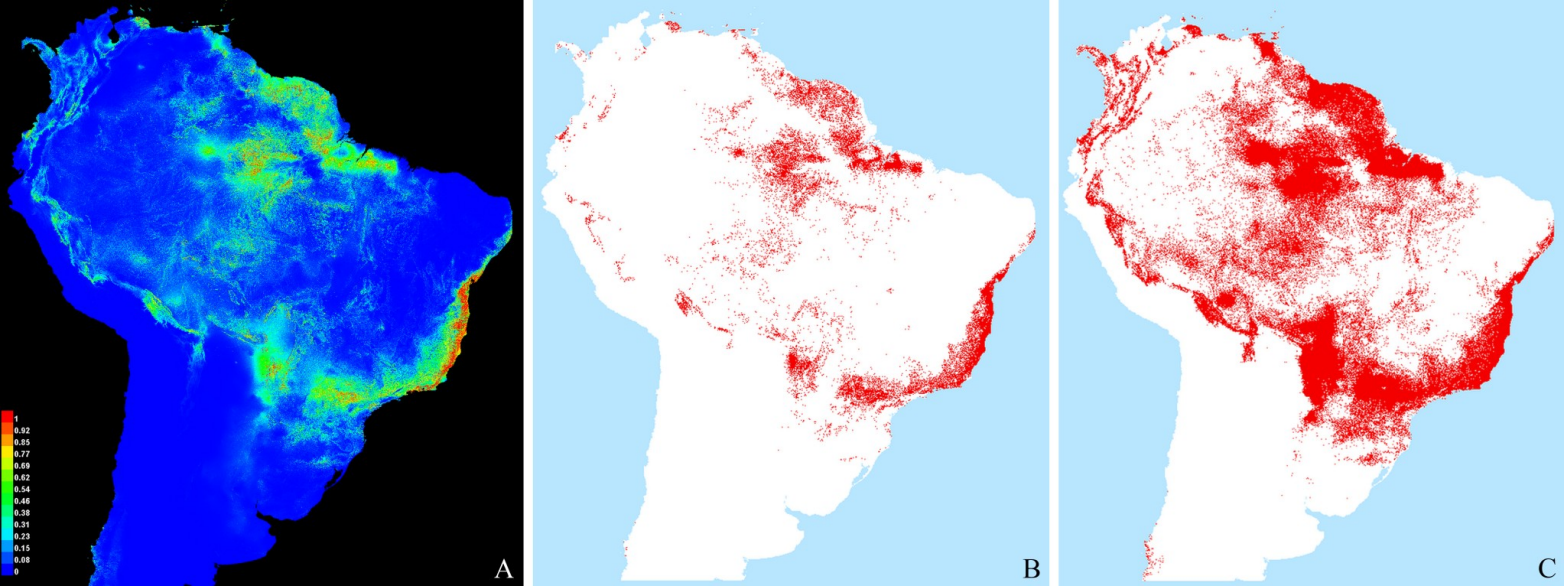
Potential distribution for *Cryptoxenodon metamorphus* sp. nov. (A) MaxEnt model with scale of probabilities. (B) Binary map with a limiar of 0.4043 (Maximum Training Sensitivity Plus Specific). (C) Binary map with a limiar of 0.1168 (Minimum Training Presence).

**Fig 23:**
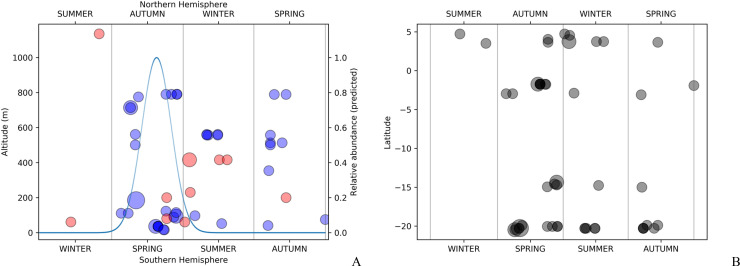
Summary of collecting records for the females of *C*. *metamorphus* sp. nov. Circles are collecting events; smallest circles = 1 specimen each, largest circle = 3 specimens. Range records plotted as two values, i.e., starting and ending dates. (A) Records grouped by altitude; red circles show specimens from the northern hemisphere. Relative abundance of specimens from the southern hemisphere shown as a best fit Gaussian curve. (B) Records grouped by latitude.

**Occurrence.** Forested areas in Amazonia (Colombia, Brazil, French Guiana) and the Atlantic Forest (Fig 21). Apparently restricted to tropical areas: no specimens have been found in samples from southern, subtropical portions of Brazil. Ecological niche modelling (Fig 22), using either a conservative (Fig 22B) or inclusive threshold (Fig 22C), shows that the species appears to be mostly concentrated in two main areas, one in central + northern Amazonia (themselves somewhat isolated by a gap), and another ranging through most of the Eastern coast, in the Atlantic Forest, and reaching Paraguay. The seasonal records of all known females (Fig 23) indicate that flying adults in the southern hemisphere occur with similar frequency in all seasons except winter (Fig 23A), with an occurrence peak apparently in mid spring. The abundance of the species also seems quite uniform in different altitudes (Fig 23A) or latitudes (Fig 23B).

## Supporting information

S1 File Full set of RGB values sampled from the female specimens. (XLSX)

S2 File Full data matrices (discrete and combined data) used in the phylogenetic analyses. (ZIP)

S3 File Maximum likelihood phylogeny of Cryptini, complete tree. (TRE)

S1 Appendix (DOCX)
