# Supplementary material for: Species delimitation, environmental cline and phylogeny for a new Neotropical genus of Cryptinae (Ichneumonidae)
Source: PLoS One. 2020 Oct 9;15(10):e0237233. doi: 10.1371/journal.pone.0237233 (PMC7546512; doi:10.1371/journal.pone.0237233)
Supplement: S1 Appendix — (DOCX) [file pone.0237233.s004.docx]

**S1 Appendix**

Full set (252 values) of measurements, in mm, from which all morphometric ratios and values in the text were derived. They are presented below as Python lists but can also be easily formatted for other programming languages. Measurements in each list follow the same order as described in Material and Methods.

Cmetamorphus = [['FAS1127', 0.22, 0.21, 0.22, 0.28, 0.50, 1.43, 26, 9.07, 0.18, 0.48, 0.51, 0.17, 9, 2.09, 2.00, 1.23, 0.46, 0.26, 0.26, 1.32, 3.99, 0.65, 4.90, 5, 0.37, 0.34, 1.64, 0.92, 0.15, 0.19, 2.03, 1.04, 0.35, 1.86, 2.05, 1.18], ['FAS4604', 0.20, 0.20, 0.22, 0.22, 0.46, 1.31, 28, 8.10, 0.18, 0.30, 0.49, 0.16, 8, 1.82, 1.86, 1.11, 0.44, 0.24, 0.20, 0.86, 3.88, 1.06, 4.25, 7, 0.35, 0.26, 1.42, 0.74, 0.12, 0.20, 1.90, 0.86, 0.33, 1.56, 1.84, 0.94], ['FAS4607', 0.12, 0.14, 0.17, 0.14, 0.33, 0.94, 24, 5.85, 0.12, 0.22, 0.32, 0.09, 6, 1.15, 1.18, 0.82, 0.28, 0.16, 0.17, 0.62, 2.75, 0.49, 3.24, 8, 0.22, 0.24, 1.01, 0.49, 0.07, 0.10, 1.16, 0.53, 0.20, 1.22, 1.31, 0.67], ['FAS4601', 0.15, 0.15, 0.21, 0.20, 0.52, 1.53, 26, 8.60, 0.14, 0.32, 0.48, 0.11, 8, 2.12, 2.09, 1.28, 0.52, 0.29, 0.24, 1.03, 4.14, 0.86, 4.74, 8, 0.38, 0.33, 1.66, 0.88, 0.15, 0.24, 1.84, 1.07, 0.39, 1.72, 2.28, 1.22], ['FAS4605', 0.20, 0.17, 0.18, 0.23, 0.44, 1.42, 27, 8.25, 0.22, 0.35, 0.43, 0.13, 7, 1.74, 1.76, 1.09, 0.39, 0.23, 0.25, 0.89, 4.65, 1.40, 4.37, 7, 0.33, 0.31, 1.34, 0.79, 0.12, 0.20, 1.72, 0.81, 0.38, 1.66, 1.64, 0.94], ['UFES49176', 0.18, 0.16, 0.20, 0.21, 0.43, 1.14, 25, 7.20, 0.13, 0.32, 0.40, 0.15, 6, 1.56, 1.54, 0.93, 0.34, 0.19, 0.18, 0.94, 3.15, 0.64, 3.99, 7, 0.29, 0.28, 1.30, 0.66, 0.12, 0.14, 1.62, 0.76, 0.27, 1.47, 1.50, 0.85], ['FAS1130', 0.20, 0.19, 0.16, 0.25, 0.54, 1.40, 27, 9.25, 0.14, 0.48, 0.53, 0.19, 8, 2.31, 2.13, 1.28, 0.50, 0.27, 0.28, 1.72, 4.45, 1.10, 4.80, 6, 0.43, 0.32, 1.70, 0.95, 0.11, 0.17, 1.48, 0.85, 0.31, 1.80, 2.03, 1.00]]
